# Whole-plant spatiotemporal responses to salinity reveal osmotic and ionic drivers of salt tolerance in broccoli

**DOI:** 10.1186/s12870-026-09390-0

**Published:** 2026-06-30

**Authors:** Juan Nicolas-Espinosa, Angel L. Guarnizo, Micaela Carvajal, Lucia Yepes-Molina

**Affiliations:** 1 Aquaporins Group, Centro de Edafologia y Biologia Aplicada del Segura, CEBAS-CSIC, Campus de Espinardo 25, Murcia, 30100 Spain; 2 Departamento Biología Vegetal, Facultad de Biología, CEIR Campus Mare Nostrum (CMN), Universidad de Murcia, Campus de Espinardo, Murcia, 30100 Spain

**Keywords:** Abiotic stress, *Brassica oleracea*, Ion homeostasis, RNA-seq, Salt tolerance mechanisms, Spatiotemporal regulation

## Abstract

**Background:**

Soil salinity is a critical threat to global agriculture and understanding the dynamic and tissue-specific responses of crops exposed to salt is essential for developing mitigation strategies.

Broccoli (*Brassica oleracea* L. var. *italica*), a crop that shows sensitivity to salinity while retaining the ability to activate adaptive responses, has not been extensively studied at the molecular level under salinity. In this study, we conducted a time-course analysis combining transcriptomic, physiological, and biochemical approaches to unravel the mechanisms underlying the tolerance of broccoli to salt stress.

**Results:**

A time-course analysis combining transcriptomics, physiology, and biochemistry revealed that broccoli employs a highly coordinated systemic strategy. First, osmotic adaptation was driven by rapid and sustained osmoprotection through *P5CS* activation and *PDH1* repression, resulting in robust proline accumulation. Second, ionic stress responses were highly tissue-specific: leaves prioritized early K⁺ homeostasis, while roots acted as primary sensors, triggering early signalling and exclusion via the *SOS1/SOS3.1* pathway, *NHX1.1/NHX2* vacuolar sequestration, and a robust root-specific induction of the cation/H⁺ exchanger *CHX20*, highlighting a prioritized mechanism for pH and ionic homeostasis. Last, shoots showed oxidative stress responses via *GSTU* and *AOX* genes, complemented by mitochondrial protection in roots.

Systemic coordination was further supported by altered hormone and phenylpropanoid profiles, reflected by the late-stage accumulation of chlorogenic acid in leaves to preserve photosynthetic function. This systemic coordination was supported by a complex hormonal redistribution of ABA, JA, and IAA in roots and early SA increases in shoots. Furthermore, salinity complexly regulated secondary metabolism; despite late-stage glucobrassicin declines in leaves, early transcriptomic activation of glucosinolate biosynthesis and differential *GTR* expression suggested enhanced glucosinolate turnover and redistribution under stress.

**Conclusions:**

Our results provide a comprehensive spatiotemporal map of the response of broccoli to salinity, highlighting key candidate genes and physiological traits associated with moderate salinity acclimation, providing a framework to explore strategies for sustaining productivity in saline environments.

**Supplementary Information:**

The online version contains supplementary material available at https://doi.org/10.1186/s12870-026-09390-0.

## Background

The genus *Brassica* belongs to the Brassicaceae family and comprises nearly 100 species, many of which are cultivated worldwide as high-value vegetables, mustards, and oilseed crops.

Among them, broccoli (*Brassica oleracea* L. var. *italica*) stands out as one of the most economically relevant crops due to its global production and nutritional value. In 2021, the combined production of broccoli and cauliflower reached approximately 26,000 kilotons (http://data.un.org/), reflecting their economic significance, and importance for human diets.

Broccoli is particularly valued as a source of bioactive compounds with proven health-promoting properties, such as glucosinolates (GSLs), polyphenols, and vitamin C. Research has linked these secondary metabolites to protective effects against several diseases which contribute to the increasing global demand for broccoli [1].

Despite its widespread cultivation, broccoli production is increasingly threatened by soil degradation processes, particularly salinization. Inappropriate irrigation practices and the accumulation of salt in arable soils represent a serious constraint for sustainable cultivation.

Salinity is a major limitation not only for broccoli but for global agriculture. Projections indicate that by 2050, up to 50% of arable land could be salinized, with devastating consequences for crops, including growth inhibition, yield losses, and even plant death under severe conditions [2]. Salt stress is a multifaceted abiotic constraint that imposes osmotic stress, ionic toxicity, and oxidative damage on plants [3].

Initially, high salt concentrations in the soil induce osmotic stress due to the reduced water potential surrounding root area, limiting root water uptake. The immediate consequence is a reduction of cell expansion, stomatal closure, and inhibition of photosynthesis. To counteract these effects, plants accumulate compatible solutes such as proline, glycine betaine, and soluble sugars, which act as osmoprotectants and stabilize cellular structures. In *Brassica* species, and particularly in broccoli, proline accumulation has been widely reported under saline conditions especially in tolerant varieties, so it is considered a reliable physiological marker of salt stress adaptation [4].

Furthermore, ionic stress is highly important in plants grown under salinity since it produces a nutrient imbalance and ion toxicity, primarily due to sodium (Na^+^) but also chloride (Cl^−^) accumulation [3]. Elevated Na^+^ concentrations disrupt potassium (K^+^)-dependent processes, destabilize enzymes, and compete with essential elements like Ca^2+^, Mg^2+^, N and P. Salt-tolerant genotypes typically maintain higher K^+^ selectivity and a favourable K^+^/Na^+^ ratio under salinity through mechanisms that exclude Na^+^ from the cytosol or compartmentalize it into vacuoles [3].

In *Brassica* crops, this ability to sustain ion homeostasis is strongly correlated with salinity tolerance [5]. Specifically, broccoli cultivars that retain more K⁺ under salt stress show improved growth and physiological performance [6], highlighting K^+^ retention as a key adaptive trait under salinity. Central to this regulation is the SOS (Salt Overly Sensitive) pathway, which controls Na^+^ efflux, as well as vacuolar Na^+^/H^+^ exchangers (NHX) and high-affinity K^+^ transporters (HKT) [3].

A third major consequence of salt stress is oxidative stress, driven by the excessive generation of Reactive Oxygen Species (ROS), including superoxide (O2.^−^), hydrogen peroxide (H_2_O_2_), and hydroxyl radicals (·OH). While ROS play signalling roles at moderate concentrations, for example, in ABA-mediated stomatal closure, their excessive accumulation damages lipids, proteins, and nucleic acids, ultimately compromising cell viability [7]. To mitigate oxidative stress, plants activate complex antioxidant defense systems comprising enzymatic components (superoxide dismutase (SOD), catalase (CAT), ascorbate peroxidase (APX), and glutathione S-transferase (GST)) along with non-enzymatic molecules (ascorbate, carotenoids, and phenylpropanoids). In addition, *Brassicaceae* species are characterized by the presence of GSLs, whose levels have been reported to increase under abiotic stress [8]. Their degradation products, such as isothiocyanates, have recognized antioxidant properties and are proposed to participate in stress-related signalling pathways. Comparative studies among *Brassica* species suggest a link between enhanced salinity tolerance, higher glucosinolate content, and increased expression of glucosinolate transporter genes (GTRs) [9].

Although several physiological responses of broccoli to salinity regarding osmotic adjustment, ion homeostasis, and antioxidant activation, have been partially described [4, 10], the underlying transcriptional regulation of these responses remains insufficiently understood. The advent of RNA-sequencing (RNA-seq) has enabled high-resolution analyses of gene expression dynamics under stress, revealing tissue-specific and time dependent responses in related species such as *Brassica napus* [11]. In a recent transcriptomic study in broccoli, we identified general patterns of gene expression under salt stress and revealed the existence of distinct temporal dynamics between leaf and root tissues [12]. These findings have highlighted the importance of temporal regulation, as the timing coordination of stress responses often determine the outcome of tolerance or susceptibility to salinity. In the present work, we build upon this dataset [12] by performing a deeper integrative analysis in combination with new physiological and biochemical measurements, aiming to provide a more comprehensive understanding of the mechanisms underlying salinity responses in broccoli.

The objective was to investigate the temporal response of broccoli to salinity by integrating physiological, metabolic, and transcriptomic perspectives. This study represents the first high-resolution, spatiotemporal analysis of the broccoli response to salt stress, focusing on the remodelling of gene expression programs related to osmotic adjustment, ion homeostasis, oxidative stress defense, and secondary metabolism at three time points: 6 h, 3 days, and 10 days after NaCl exposure. By combining high-resolution RNA-seq with detailed physiological measurements, we provide a time-resolved view of the coordinated responses. This integrative and time-resolved approach seeks to identify the key molecular and metabolic mechanisms underlying Na^+^ and Cl^−^ toxicity, with particular emphasis on their time-dependent regulation, helping to clarify the molecular basis of salt stress adaptation in broccoli.

## Methods

### Plant materials and application of salt stress

Seeds of *Brassica oleracea* L. var. *italica* cv. Parthenon (broccoli) (SAKATA seed Ibérica, Alboraya, Valencia, Spain) were pre-hydrated in deionized water for 24 h and germinated in darkness at 28 °C for two days using vermiculite as substrate. Uniformly germinated seedlings were transferred to hydroponic systems containing full Hoagland solution (6 mM KNO_3_, 4 mM Ca(NO_3_)_2_, 2 mM NH_4_H_2_PO_4_, 1 mM MgSO_4_, 25 µM H_3_BO_3_, 4 µM MnSO_4_, 4 µM ZnSO_4_, 1 µM CuSO_4_, 0.13 µM (NH_4_)_6_Mo_7_O_24_, and 40 µM Fe^3+^-EDDHA). The plants were grown in a controlled-environment chamber under a 16-h light and 8-h dark photoperiod, with day/night temperatures of 25/20 °C, relative humidity 60–80% and photosynthetically active radiation (PAR) of 400 µmol m^− 2^ s^− 1^, provided by a Pacific LED, WT 470 C, LED8OS/840 PSD WB L1600, Philips.

The experiment was conducted using sixteen plastic containers, each initially filled with four broccoli plants growing under control conditions. After 15 days of acclimation, 80 mM NaCl was applied to the nutrient solution of the eight randomly designated containers, while the remaining containers continued under control conditions. Each container was considered an independent experimental unit. To avoid pseudoreplication, only one plant per container was sampled at each time point, resulting in *n* = 8 independent biological replicates per treatment and time point. Plant samples were harvested at 6 h, 3 days, and 10 days after salinity application.

### Physiological and chemical measurements

#### Biomass

At each of the three sampling time points (6 h, 3 days, and 10 days after salt stress), eight control and eight salt-treated plants were harvested and weighed to determine their fresh weight (FW).

#### Photosynthetic and chlorophyll fluorescence measurements

Leaf gas exchange measurements were performed on 8 to 16 plants per treatment at each time point: 6 h, 3 days, and 10 days after the application of NaCl. Measurements were taken on fully expanded young leaves using a LI-6800 Portable Photosynthesis System (LI-COR^®^, Inc., Lincoln, NE, USA) by the Plant Biotechnology Service (ACTI) at the University of Murcia (Spain). Additionally, chlorophyll fluorescence parameters were measured using a Chlorophyll Fluorometer OS30P+ (Opti-Sciences, Inc., Hudson, NH, USA), to assess photosynthetic performance, particularly parameters commonly associated with photosystem II (PSII) activity.

The following variables were evaluated: maximum/potential quantum efficiency of photosystems (F_v_/Fₘ), maximum primary yield of photosystems photochemistry (F_v_/F₀), photosynthetic performance index (PI), and electron transport flux per cross section (ETo/CS).

#### Analysis of mineral nutrients

The quantification of macro- and micronutrient concentrations in plant tissues was performed at the Ionomics Laboratory (CEBAS-CSIC, Murcia, Spain), following the ISO 11.885 (1996) standards. Analyses were carried out using Inductively Coupled Plasma–Optical Emission Spectrometry (ICP-OES) on a Thermo ICAP 6500 Duo instrument (Thermo Fisher Scientific, Waltham, MA, USA). The nutrient concentrations were expressed as gkg^− 1^ dry weight (DW).

Roots and leaves from four independent plants per treatment and time point were analysed.

#### Secondary metabolites and proline extraction and analysis (HPLC-MS)

Root and leaf secondary metabolites and proline of four plants from each treatment and time were analysed. The extraction and analysis of GSLs and phenolic compounds were performed as described in Albaladejo-Marico et al. [13]. 100 mg of lyophilized powder was extracted with 1 mL of 70% methanol in water (CH_3_OH: H_2_O; 1:10). The extraction process was carried out in a bath at 72 °C for 30 min, with vortexing every 5 min. Next, the samples were cooled on ice and subsequently centrifuged at 10,000 × g for 15 min. Supernatant was collected and filtered through a 0.22 μm diameter Millex Syringe Filter, Durapore (PVDF) by Merck Millipore (Billerica, MA, USA).

For phytohormone extraction, 100 mg of deep-frozen ground leaf or root samples (− 80 °C) were extracted with 500 µL of methanol: isopropanol: acetic acid (25:24.5:0.5) and sonicated for 30 min, with vortexing every 10 min. Subsequently, the samples were centrifuged at 16,000 × g for 5 min at 4 °C. The supernatant was collected, and the pellets were re-extracted with 250 µL of the same solvent solution. Finally, the supernatants were collected and filtered through a 0.22 μm pore diameter Millex Syringe filter, Durapore (PVDF) by Merck Millipore (Billerica, MA, USA).

Proline was extracted from 50 mg of deep-frozen ground leaf or root samples (− 80 °C) using 1 mL of ethanol: Milli-Q water (40:60, v/v), followed by overnight incubation at 4 °C. Subsequently, the samples were centrifuged at 14,000 × g for 5 min at 4 °C.

The identification and quantification analysis were conducted using High-Performance Liquid Chromatography - Mass Spectrometry (HPLC-MS) at the Metabolomic and Proteomic Service (ACTI - Murcia University, Spain). For all the analyses, the procedure was carried out in negative ion mode (for GSLs, phenolic compounds, and phytohormones) and in positive ion mode (for proline analysis) on an Agilent 1290 Series II HPLC (Agilent Technologies, Santa Clara, CA, USA) connected to an Agilent iFunnel 6550 Quadrupole Time-of-Flight (Q-TOF) Mass Spectrometer (Agilent Technologies, Santa Clara, CA, USA) equipped with an Agilent Jet Stream (AJS) Dual Electrospray Ionization (ESI). Chromatographic separation was performed on a reversed-phase C18 column (Zorbax Eclipse Plus, 2.1 × 100 mm, 1.8 μm) using water and organic solvents acidified with formic or acetic acid as mobile phases, following gradient conditions similar to those previously described [13]. Quantification was performed using external standards, including sinigrin and glucoraphanin (GRA) (MedChemExpress) for aliphatic GSLs; glucobrassicin (GBA) (Phytoplan) for indole GSLs; and chlorogenic, caffeic, and sinapic acids (Sigma-Aldrich) for phenolic compounds. The hormones analyzed, abscisic acid (ABA), jasmonic acid (JA), methyl jasmonate (MeJA), salicylic acid (SA) (Sigma-Aldrich), indole-3-acetic acid (IAA), and zeatin (MedChemExpress), were selected as a targeted panel covering representative stress-related hormones and auxin- and cytokinin-associated growth markers involved in salinity responses [14]. All targeted metabolites were analyzed in both root and leaf tissues. However, due to the limited biomass of root under salt stress and the concentrations being below the limit of quantification (LOQ) for several compounds, only the quantifiable data from the shoot are presented for these specific metabolites. Metabolite concentrations were normalized to tissue biomass and are expressed as nmol g^− 1^ DW for all analyzed compounds.

### RNA sequencing

Root and leaf samples from four plants were harvested 6 h, 3 days, and 10 days after salt treatment, or from control plants (as described above). Total RNA was extracted using the NZY Total RNA Isolation kit (NZYtech, Lisbon, Portugal) and RNA integrity was assessed using an Agilent Bioanalyzer 2100 system (Agilent Technologies) with a 2100 expert Eukaryote total RNA Nano Chip. Library preparation and transcriptome sequencing were performed at the Molecular Biology Service (ACTI, Murcia University, Spain) using NextSeq 1000/2000 P3 (200 cycles) with 75 bp paired-end reads (minimum of 25 million reads per sample) following the protocols previously described in detail by Yepes-Molina et al. [12].

The Illumina RNA-seq raw reads used in this study were originally generated and described in Yepes-Molina et al. [12]. This transcriptomic dataset is deposited in the NCBI database under BioProject accession number PRJNA1327109. Data processing followed the same bioinformatic pipeline detailed in that previous work. The broccoli gene codes (BoL) were previously identified and functionally annotated based on Arabidopsis orthologs using Dicots PLAZA 5.0 Database. Gene functions and annotations were retrieved from the *TAIR* and *Ensembl Plants* databases, and used to generate the functional gene lists for downstream analyses. Heatmaps of targeted pathways were generated using the *ComplexHeatmap* R package (v 1.20.0) based on genes showing significant differential expression (q-value ≤ 0.05) to capture moderate but biologically relevant shifts in specialized metabolism.

### Statistical analysis

Statistical analyses and data presentation were conducted using Origin(Pro), Version 2024 (OriginLab Corporation, Northampton, MA, USA). Tests were preceded by a normality test and a Grubbs’ test to identify potential outliers. Pairwise comparisons between control and salt-treated plants at each time point were performed using Student’s t-test. All presented values represent means ± SE. R packages FactoMineR and factoextra were used to generate Principal Component Analysis (PCA).

## Results

### Physiological time-course response of broccoli plants to salinity

**Fig. 1:**
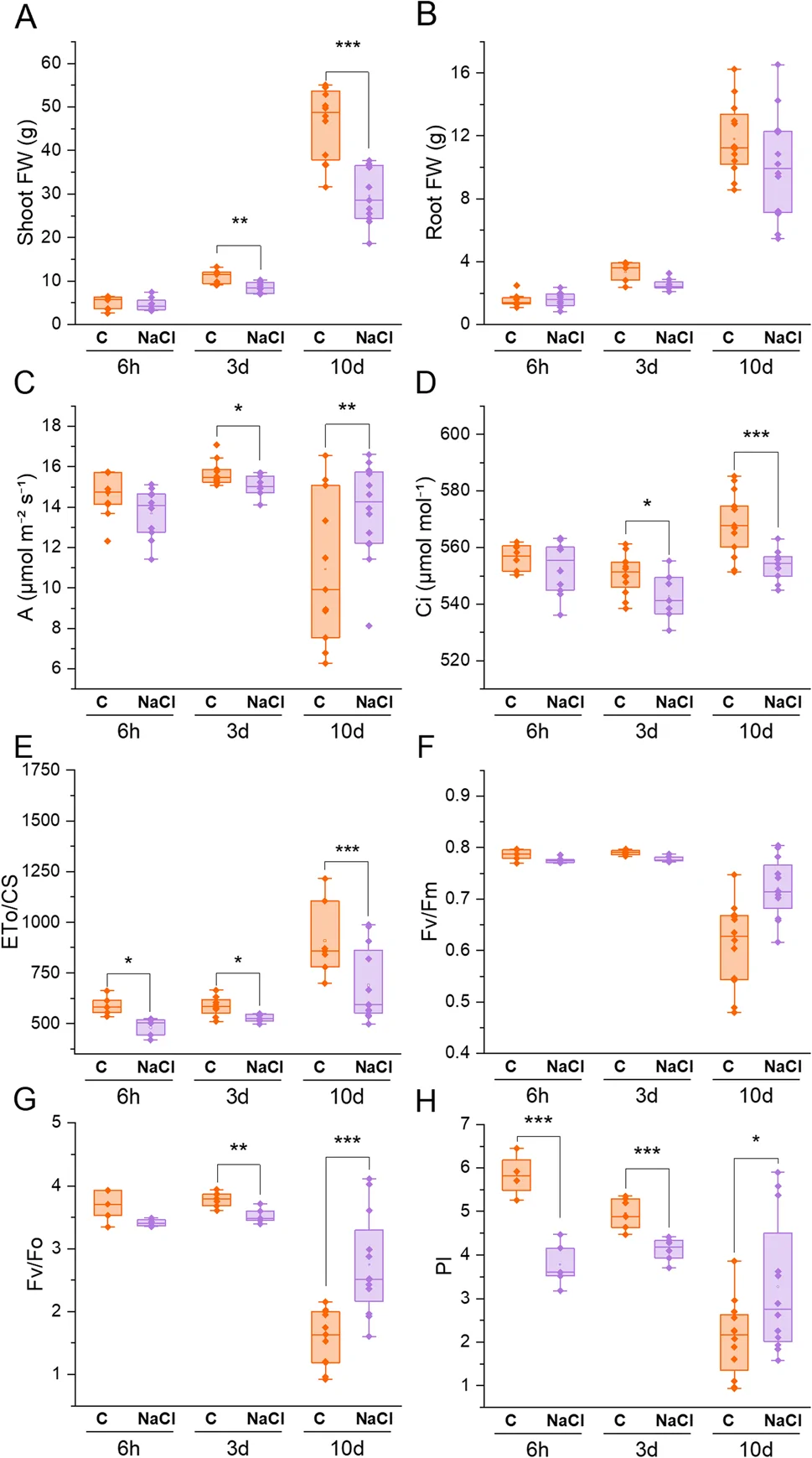
Weight and photosynthesis parameters in broccoli plants. Different parameters measured in broccoli plants that were grown for 15 days under control conditions (C) prior to the onset of salinity stress (NaCl). Sampling was conducted at 6 h, 3 d, and 10 d after treatment application: (**A**) Shoot fresh weight (FW, g), (**B**) root FW (g), (**C**) net photosynthesis rate (A, µmol m^− 2^ s^− 1^), (**D**) calculated substomatal CO_2_ concentration (Ci, µmol mol-1), (**E**) electron transport flux per cross section (ET_0_/CS), (**F**) maximum/potential quantum efficiency of photosystems (Fv/Fm), (**G**) maximum primary yield of photosystems photochemistry (Fv/F0), (**H**) photosynthetic performance index (PI). Data are represented as boxes (25–75%), error bars represent the range within a 1.5 quartile, the horizontal line within each box represents the median, while the white square marker indicates the mean value (*n* = 6 independent biological replicates, with each plant harvested from a separate hydroponic unit). Asterisks mean statistical differences after Student’s t-test (*p < 0.05, **p < 0.01, ***p < 0.001)

The temporal response of broccoli plants to high salinity stress revealed a progressive physiological adjustment across time points (6 h, 3 d, and 10 d) (Fig. 1; Additional file 1).

Total FW of the broccoli plants significantly declined from 3 days onwards and further decreased at 10 days, resulting in an overall reduction of 30% (Additional file 1) compared to control broccoli plants. A similar pattern was observed in the shoot FW of salt-treated plants (Fig. 1A), whereas no significant differences were detected in root FW throughout the experiment (Fig. 1B). Regarding plant responses in CO_2_ exchange parameters, A, decreased at 3 days under salinity by 30–40% but showed a partial recovery at 10 days compared to the corresponding controls. In contrast, internal concentration of CO_2_ (Ci) showed under saline conditions significant reductions at both 3 and 10 days, decreasing by 20–30% (Fig. 1C-D). The efficiency of electron transport (ET_0_/CS) was consistently lower in salt-treated plants across all sampling times, with reduction of 20–30% (Fig. 1E). Similarly to A, chlorophyll fluorescence parameters Fv/F0 showed a decline under saline conditions by 20% at 3 days but increased at 10 days (Fig. 1G). However, performance index (PI) showed a strong early reduction, decreasing by 40% at 6 h and 10% at 3 days of salinity application, and an increase by 30% at 10 days (Fig. 1H).

**Fig. 2:**
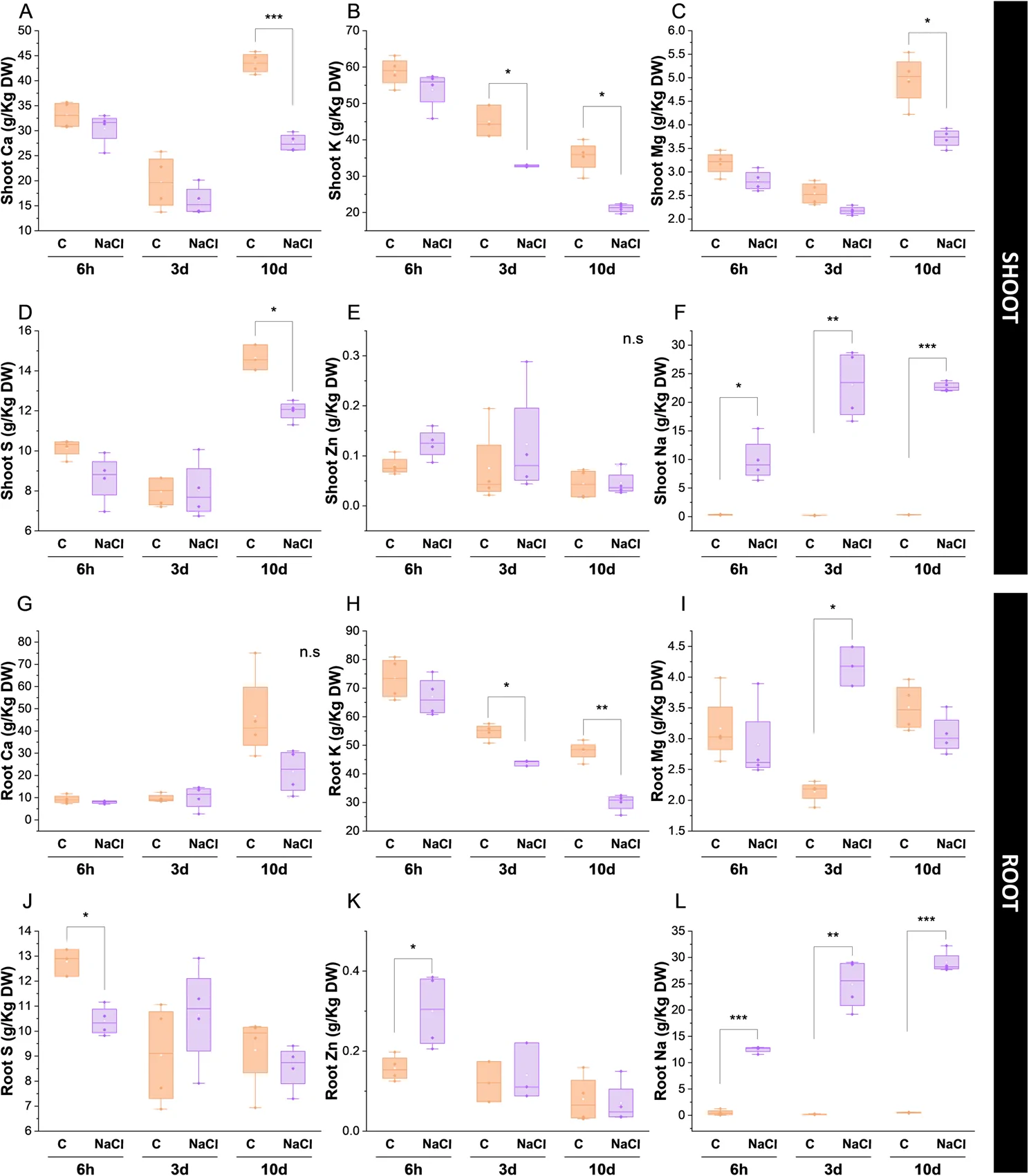
Analysis of mineral nutrients in broccoli plants. Different minerals were measured in broccoli plants that were grown for 15 days under control conditions (C) prior to the onset of salinity stress (NaCl). Sampling was conducted at 6 h, 3 d, and 10 d after treatment application: (**A**) shoot Ca (g/Kg DW), (**B**) shoot K (g/Kg DW), (**C**) shoot Mg (g/Kg DW), (**D**) shoot S (g/Kg DW), (**E**) shoot Zn (g/kg DW), (**F**) shoot Na (g/Kg DW), (**G**) root Ca (g/Kg DW), (**H**) root K (g/Kg DW), (**I**) root Mg (g/Kg DW), (J) root S (g/Kg DW), (**K**) root Zn (g/kg DW), and (**L**) root Na (g/Kg DW). Data are represented as boxes (25–75%), error bars represent the range within a 1.5 quartile, the horizontal line within each box represents the median, while the white square marker indicates the mean value (*n* = 4 independent biological replicates, with each plant harvested from a separate hydroponic unit). Asterisks mean statistical differences after Student’s t-test (*p < 0.05, **p < 0.01, ***p < 0.001)

Salinity induced a clear reorganization of nutrient profiles, as revealed by PCA, which showed distinct separation between treatments and time points in both leaves and roots (Fig. 2; Additional files 2–4). A progressive decline in K levels was evident throughout plant development in both tissues. Specifically, salinity-treated plants showing significantly lower K levels starting from the third day of the experiment, with reductions ranging from 20% to 40% depending on the tissue and time point (Fig. 2B and H). Concurrently, salt stress triggered a massive accumulation of Na during all analysed time points, with concentrations reaching up to 25 g/Kg (Fig. 2F and L). In shoot, Ca, Mg, and S vectors point in the opposite direction, aligning more closely with the control samples indicating a relative depletion of these elements under salt treatment, which accounted for a 15% (S), 25% (Mg) and 35% (Ca) reduction (Additional files 2A, Fig. 2A, C and D). In roots, a similar nutrient-driven separation is observed, with root nutrient changes most pronounced at six hours, where a decrease in S (by 20%) and an increase in Zn (by 100%) was observed (Additional files 2B, Fig. 2J-K). Overall, these results indicate a time-dependent and tissue-specific regulation of mineral homeostasis under salinity stress, driven by shifts in the relative contribution of individual mineral elements.

### Biochemical time-course response of broccoli plants to salinity

**Fig. 3:**
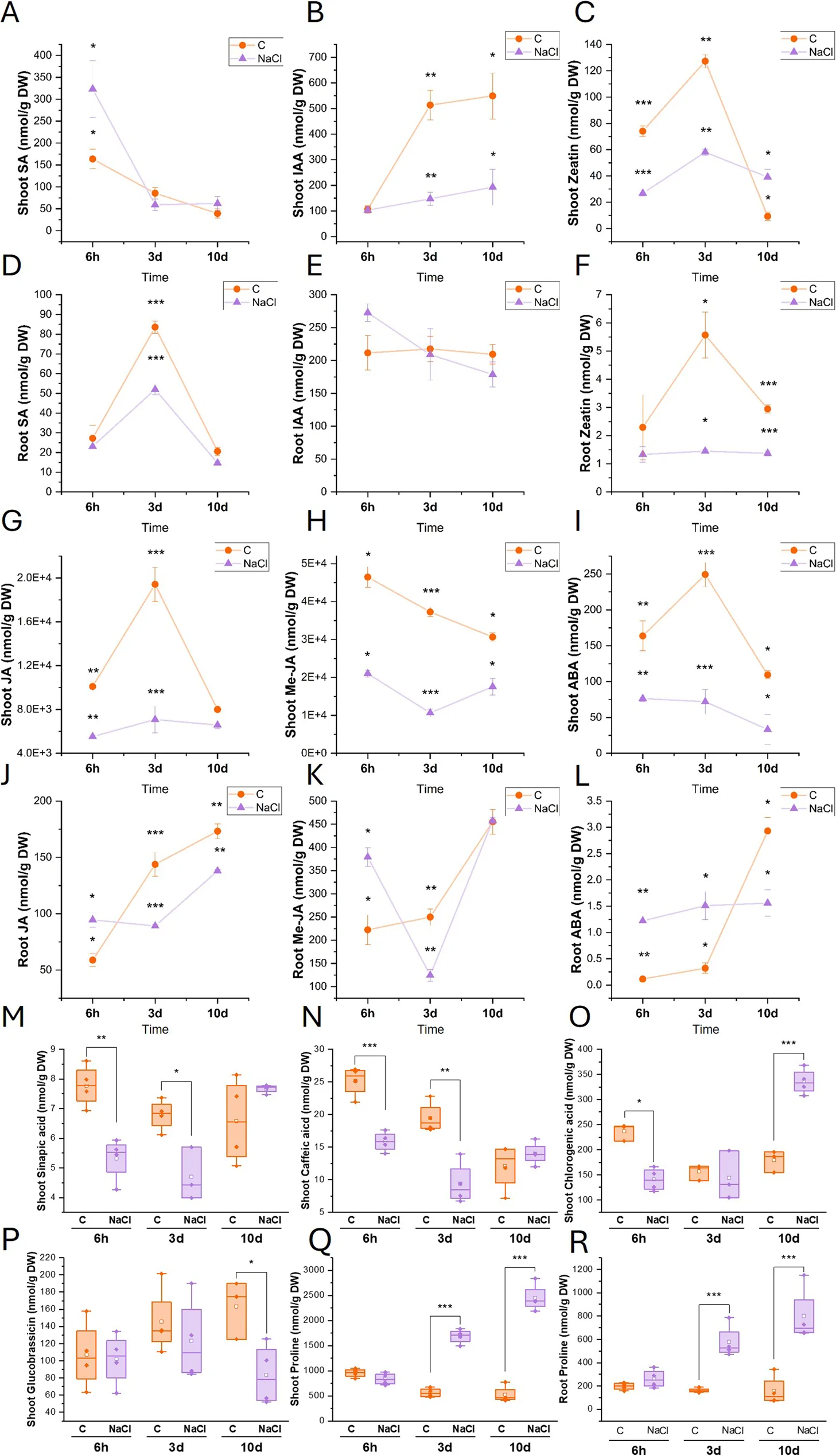
Analysis of secondary metabolites in broccoli plants. Metabolites (nmol g⁻¹ DW) measured in broccoli plants that were grown for 15 days under control conditions (**C**) prior to the onset of salinity stress (NaCl). Sampling was conducted at 6 h, 3 d, and 10 d after treatment application: salicylic acid (SA) in (**A**) leaf and (**D**) root, indole acetic acid (IAA) in (**B**) leaf and (**E**) root, zeatin in (**C**) leaf and (**F**) root, jasmonic acid (JA) in (**G**) leaf and (**J**) root, methyl jasmonate (MeJA) in (**H**) leaf and (**K**) root, abscisic acid (ABA) in (**I**) leaf and (**L**) root, (**M**) leaf sinapic acid, (**N**) leaf caffeic acid, (**O**) leaf chlorogenic acid, (**P**) leaf glucobrassicin, and proline in (**Q**) leaf and (**R**) root. Data are represented as mean ± SE (*n* = 4 independent biological replicates, with each plant harvested from a separate hydroponic unit) or as boxes (25–75%), error bars represent the range within a 1.5 quartile, the horizontal line within each box represents the median, while the white square marker indicates the mean value (*n* = 4 independent biological replicates, with each plant harvested from a separate hydroponic unit). Asterisks mean statistical differences after Student’s t-test (*p < 0.05, **p < 0.01, ***p < 0.001)

The biochemical response of broccoli to salinity was assessed through the quantification of key metabolites, including phytohormones, GSLs, and phenolic compounds (Fig. 3). In leaves, a general decrease in the levels of jasmonic acid (JA), methyl jasmonate (MeJA), and abscisic acid (ABA) was observed in response to salt stress starting at 6 h and persisting throughout the experiment, with reductions ranging between 50% and 72% compared to controls (Fig. 3G**-** I).

Indole-3-acetic acid (IAA) at later sampling points was reduced under salinity by approximately 70% at 3 d and 65% at 10 d compared with the corresponding control plants (Fig. 3B). In contrast, a 2-fold increase in salicylic acid (SA) was observed only 6 h after treatment, indicating an early but non-sustained response to salinity (Fig. 3A).

In roots, hormone dynamics followed a distinct temporal pattern. JA, MeJA, and ABA content increased at 6 h in response to salinity by 58%, 72%, and 6-fold, respectively, but progressively declined at later time points (Fig. 3J**-** L). SA and zeatin levels, in particular, exhibited a marked reduction from the day 3 onwards, with SA levels decreasing by 38% (Fig. 3D) and zeatin levels showing up to a 74% decrease compared to controls (Fig. 3F). Salinity modified ABA accumulation relative to the corresponding control plants at each sampling point; ABA levels were higher under salinity than in control plants at 6 h and 3 d, with increases of approximately 6-fold and 5-fold, respectively. However, at 10 d this pattern was reversed, with ABA levels being approximately 47% lower under salinity than in control plants (Fig. 3L).

Secondary metabolites in leaves showed compound-specific responses. Sinapic and caffeic acids levels decreased in response to salinity as early as 6 h after treatment, showing reductions of approximately 32% and 36% respectively, and remained lower than controls at 3 days (Fig. 3M, N). Chlorogenic acid also showed an initial reduction of 45% at 6 h, but its levels significantly increased in salt-treated plants by 83% at 10 days (Fig. 3O). Glucobrassicin levels decreased in leaf tissue by 54% 10 days post-treatment (Fig. 3P).

Proline content was analysed in leaves (Fig. 3Q) and roots (Fig. 3R). In both tissues, proline levels increased by 4- and 5-fold at 3 and 10 days after salt treatment, respectively.

### Genes involved in different stages of salt stress in broccoli plants

To assess transcriptomic reprogramming under saline conditions, DEGs were identified and functionally classified. A comprehensive RNA-seq analysis from our previous study [12] revealed a global transcriptomic response comprising a grand total of 1914 and 1266 DEGs identified across all time points in shoot and root tissues, respectively. PCA confirmed a clear separation between control and salt-treated samples [12]. DEGs were further categorized into major stress-related functional groups, with their specific time-course proportions detailed in Additional file 5. In shoots, a total of 71 genes were associated with osmotic stress (36 up-regulated, 35 down-regulated), 11 with ionic stress, and 27 with oxidative stress across all time points, representing 3.7%, 0.6%, and 1.4% of the entire salinity-responsive shoot transcriptome, respectively (collectively accounting for 5.7% of total shoot DEGs). In roots, the response was more pronounced, with 86 genes involved in osmotic adjustment, 19 in ionic homeostasis, and 36 in oxidative stress defense, which corresponded to 6.8%, 1.5%, and 2.8% of the global root DEG pool, respectively (collectively representing 11.1% of total root DEGs). These functional categories represent a significant proportion of the salinity-induced transcriptomic reprogramming. The highest number of DEGs was detected at 6 h in both tissues, indicating a rapid early transcriptional response to salinity.

#### Osmolyte biosynthesis

**Fig. 4:**
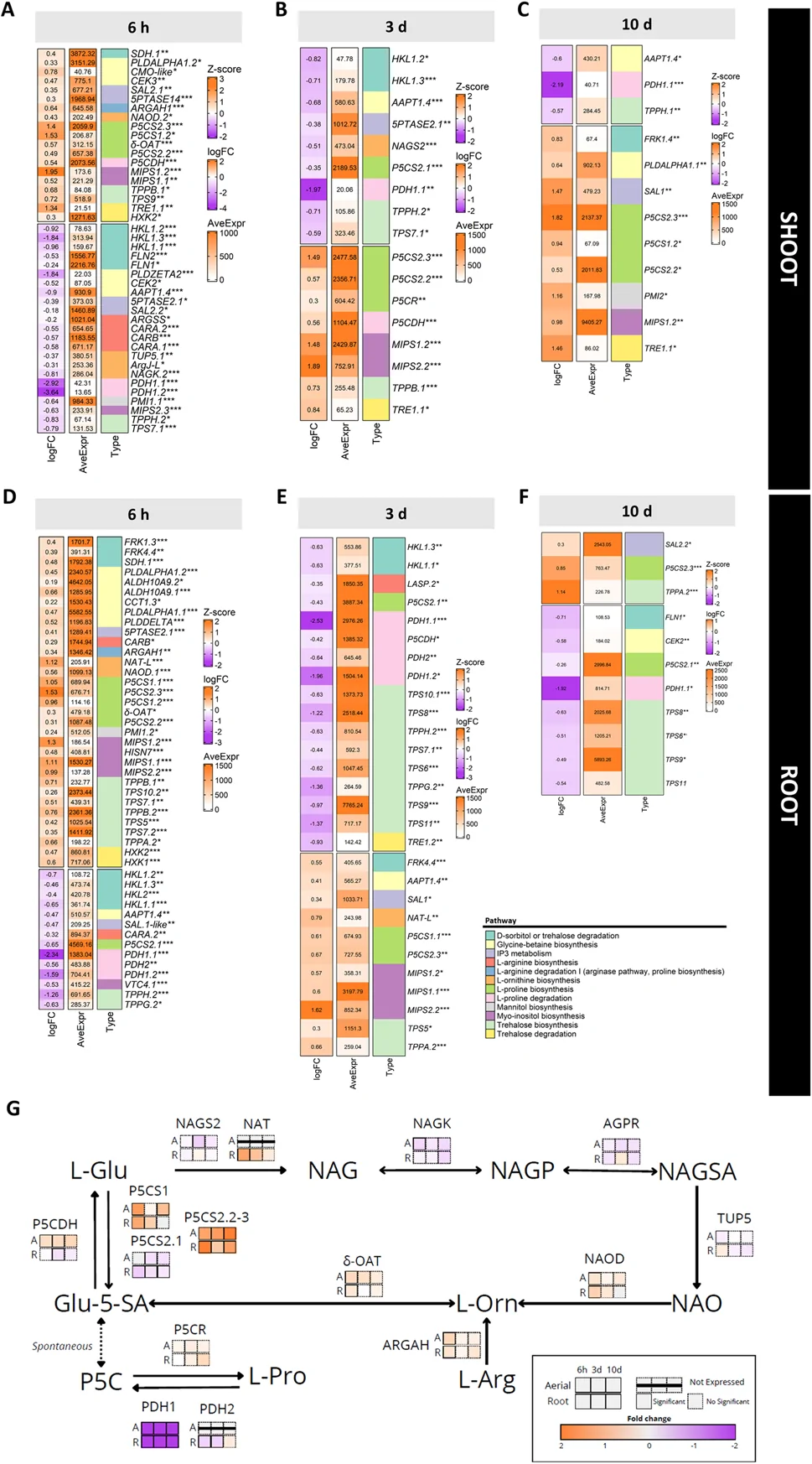
Differentially expressed genes (DEGs) involved in osmotic adjustment. Gene expression related to osmolyte biosynthesis in broccoli plants subjected to salt treatment and control conditions at 6 h (6 h), 3 days (3 d), and 10 days (10 d) after salt application, in shoot (**A**, **B**, **C**) and root (**D**, **E**, **F**). Purple and orange indicate negative and positive fold change (logFC), respectively. Mean expression (AveExpr) and gene name are shown. Statistical significance of gene expression differences between salt-treated and control plants was determined based on adjusted p-values (padj) obtained from differential expression analysis (* *p* < 0.05, ** *p* < 0.01, *** *p* < 0.001). Genes shown are grouped by the different pathways involved. **G** Proline pathway and ornithine pathway representation where the abbreviations of the compounds mean: L-glutamate (L-Glu), L-glutamate-5-semialdehyde (Glu-5-SA), (S)-1-pyrroline-5-carboxylate (P5C), L-Pro (L-proline), NAG (N-acetyl-L-glutamate), NAGP (N-acetyl-L-glutamyl-5-phosphate), NAGSA (N-acetyl-L-glutamate-5-semialdehyde), NAO (N(2)-acetyl-L-ornithine), L-Orn (L-ornithine), L-Arg (L-arginine). The abbreviations of the enzymes: P5CS1-2 (Δ1-pyrroline-5-carboxylate synthase), P5CDH (Δ1-pyrroline-5-carboxylate dehydrogenase), PDH1-2 (Proline dehydrogenase), P5CR (pyrroline-5-carboxylate reductase), NAGS2 (N-acetyl-L-glutamate synthetase), NAT (Acyl-CoA-N-acyltransferase), NAGK (N-acetyl-glutamate kinase), AGPR (N-acetyl-glutamate 5-semialdehyde dehydrogenase), TUP5 (N-acetyl-γ-glutamyl-phosphate reductase), NAOD (N-acetylornithine-δ-transaminase), δ-OAT (ornithine-δ-aminotransferase), and ARGAH (arginase)

Genes involved in osmolyte biosynthesis showed a coordinated and time-dependent response to salinity (Fig. 4 and Additional file 6). In both tissues, the most pronounced transcriptional changes occurred at 6 h, followed by a gradual reduction in the number of DEGs over time. A higher number of DEGs was detected in roots than in shoots, although the affected genes were associated with similar metabolic pathways. Notably, proline metabolism represented the dominant transcriptional response.

In leaves (Fig. 4A-C), genes involved in L-proline biosynthesis were upregulated from the early stages and remained induced throughout the experiment. Conversely, genes associated with L-proline degradation were consistently downregulated at all time points. A similar trend was observed in roots (Fig. 4D-F). Additionally, in leaves, genes involved in trehalose degradation were also upregulated, while those associated with trehalose biosynthesis were repressed. In contrast, myo-inositol biosynthesis exhibited dynamic regulation over time (Fig. 4A-C). In roots, an early activation of several genes involved in glycine betaine biosynthesis was observed, together with the induction of genes involved in myo-inositol metabolism at 6 h and 3 days (Fig. 4D-E).

To clarify the underlying response mechanisms, transcriptional changes in proline metabolism were integrated into a representative pathway scheme (Fig. 4G). The main L-proline biosynthesis pathway was upregulated at all time points, primarily driven by the consistent and significant induction of *P5CS2.2-3* (pyrroline-5-carboxylate synthetase) in both tissues, with logFC values of 0.49/1.4, 0.57/1.49, and 0.52/1.82 at each time point. Interestingly, *P5CS2.1* showed a significant downregulation specifically in the roots across all time points (with logFC values of -0.65, -0.43, and − 0.26), while no significant changes were observed in the leaves. The expression of *P5CS1* also contributed to this response, although its significant upregulation was time- and tissue- dependent. Conversely, *PDH1* (proline dehydrogenase) was strongly downregulated in both tissues with logFC values of -2.92, -1.97, and − 2.19 in leaves, and − 2.34, -2.53, and − 1.92 in roots. Whereas *PDH2* was downregulated only in roots, as no expression was detected in leaves. Additionally, at 6 h post-stress application, L-ornithine and L-arginine appeared to serve as precursors for L-proline, as evidenced by the upregulation of key enzymatic genes. Notably, *ARGAH* (arginase), which converts L-arginine into L-ornithine, and *δ-OAT* (ornithine-δ-aminotransferase), which facilitates the conversion of L-ornithine into L-glutamate-5-semialdehyde, were significantly upregulated, with logFC values of 0.64 and 0.57 in leaves and 0.34 and 0.3 in roots, respectively.

#### Ion transporters

**Fig. 5:**
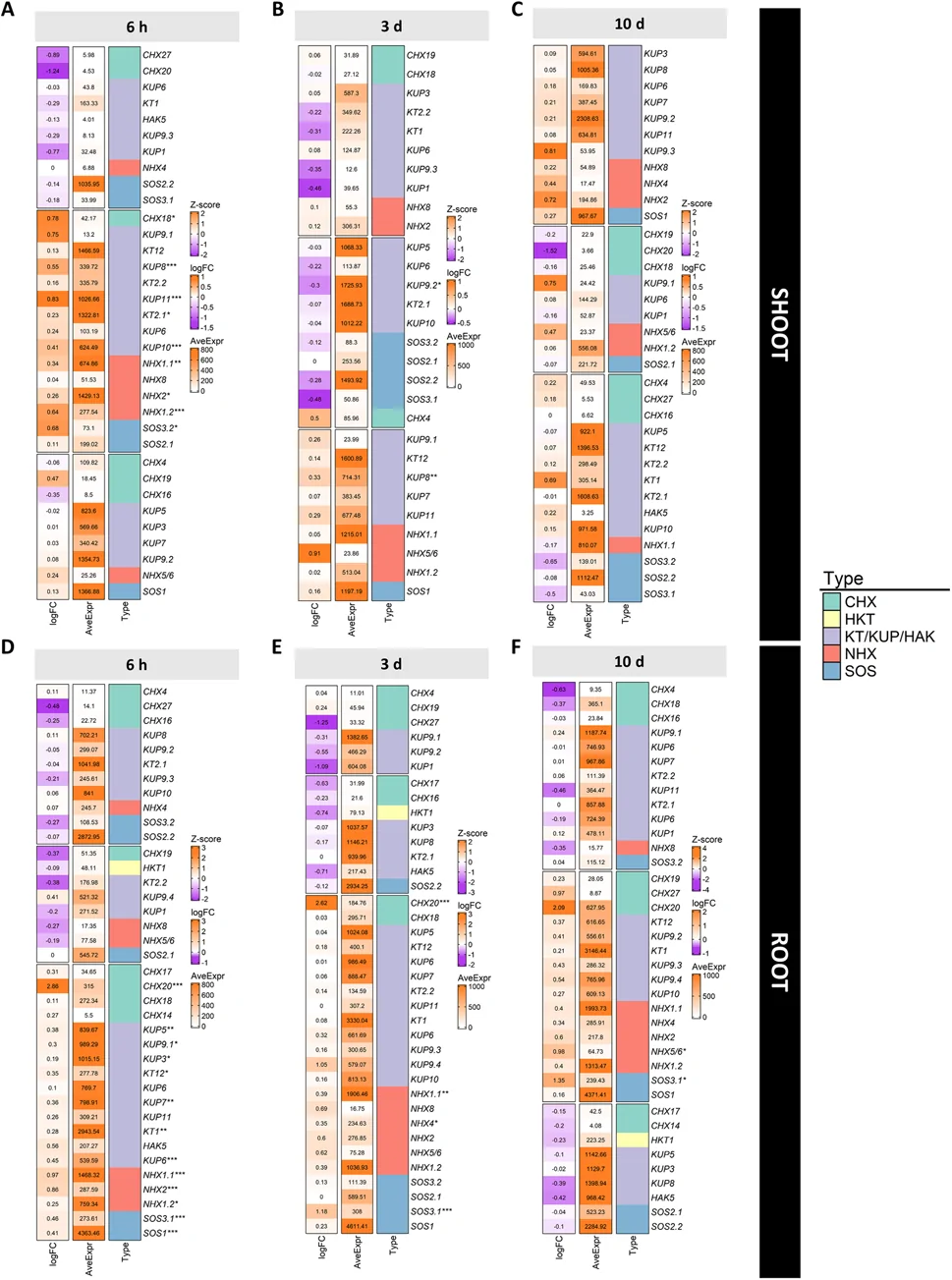
Differentially expressed genes (DEGs) implicated in ionic stress response. Ionic stress-related gene expression in salt-treated and control broccoli plants 6 h (6 h), 3 days (3 d), and 10 days (10 d) after salt application in shoot (**A**, **B**, **C**) and root (**D**, **E**, **F**). Purple and orange indicate negative and positive fold change (logFC), respectively. Mean expression (AveExpr) and gene name are shown. Statistical significance of gene expression differences between salt-treated and control plants was determined based on adjusted p-values (padj) obtained from differential expression analysis (* *p* < 0.05, ** *p* < 0.01, *** *p* < 0.001). Genes shown in the heatmaps are grouped by gene type: CHX, cation/H+ - exchanger; HKT, high-affinity K+ transporter; HAK/KUP/KT, high-affinity K+ transporters/K+ uptake permeases/K+ transporters; NHX, sodium/hydrogen antiporter; SOS, Salt Overly Sensitive proteins

The expression of genes encoding ion transporters in response to NaCl stress was analysed in broccoli plants at three time points. The analysis was conducted on both leaf and root, focusing on major transporter families involved in ion homeostasis under salt stress: CHX (cation/H^+^ - exchanger), HKT (high-affinity K^+^ transporter), HAK/KUP/KT (high-affinity K^+^ transporters/K^+^ uptake permeases/K^+^ transporters), NHX (sodium/hydrogen antiporter), and SOS (Salt Overly Sensitive) proteins (Fig. 5 and Additional file 7). The largest number of DEGs was observed 6 h after salt application, with 8 DEGs in leaves and 13 in roots. By 3 days, this number declined to 2 in leaves and 4 in roots, while at 10 days, no DEGs were detected in leaves, and only 2 DEGs were identified in roots.

In leaves, NaCl treatment induced a transient upregulation of several K⁺ transporter genes (KUP family), particularly at 6 h. Notably, *KUP11* and *KUP10* exhibited logFC of 0.83 and 0.41, with high basal expression levels of 1,026 and 624, respectively. *KUP8* showed increased expression at both 6 h and 3 days, while *KUP9.2*, which exhibited a high basal expression level of 1,725, was downregulated at 3 days. Genes involved in Na^+^ homeostasis, including *NHX1.1*,* NHX1.2*, and *NHX2*, were also upregulated at 6 h (logFC of 0.34, 0.64, and 0.26, respectively), together with *SOS3.2*, although its average expression level remained low in the leaves (Fig. 5A-C).

In roots, the response was broader and mainly detected at 6 h under salinity. This included five *KUP* genes, two *KT* genes *(KT1* and *KT2)*, three *NHX* genes, and both *SOS1* and *SOS3.1*.

Among them, *CHX20* showed the strongest and most sustained induction, with logFC of 2.66 at 6 h and 2.62 at 3 days, and its expression was predominantly restricted to roots. At 3 days, *NHX1.1* also exhibited sustained upregulation, along with *SOS3.1*, whose logFC increased from 0.46 at 6 h to 1.18 at 3 days. By 10 days, only two genes exhibited differential expression: *NHX5/6* (0.98) and *SOS3.1* (1.35). Notably, *SOS3.1* was the only gene that remained consistently upregulated throughout the experiment (logFC = 0.46, 1.18, and 1.35 at each time) (Fig. 5D-F).

#### Oxidative stress

**Fig. 6:**
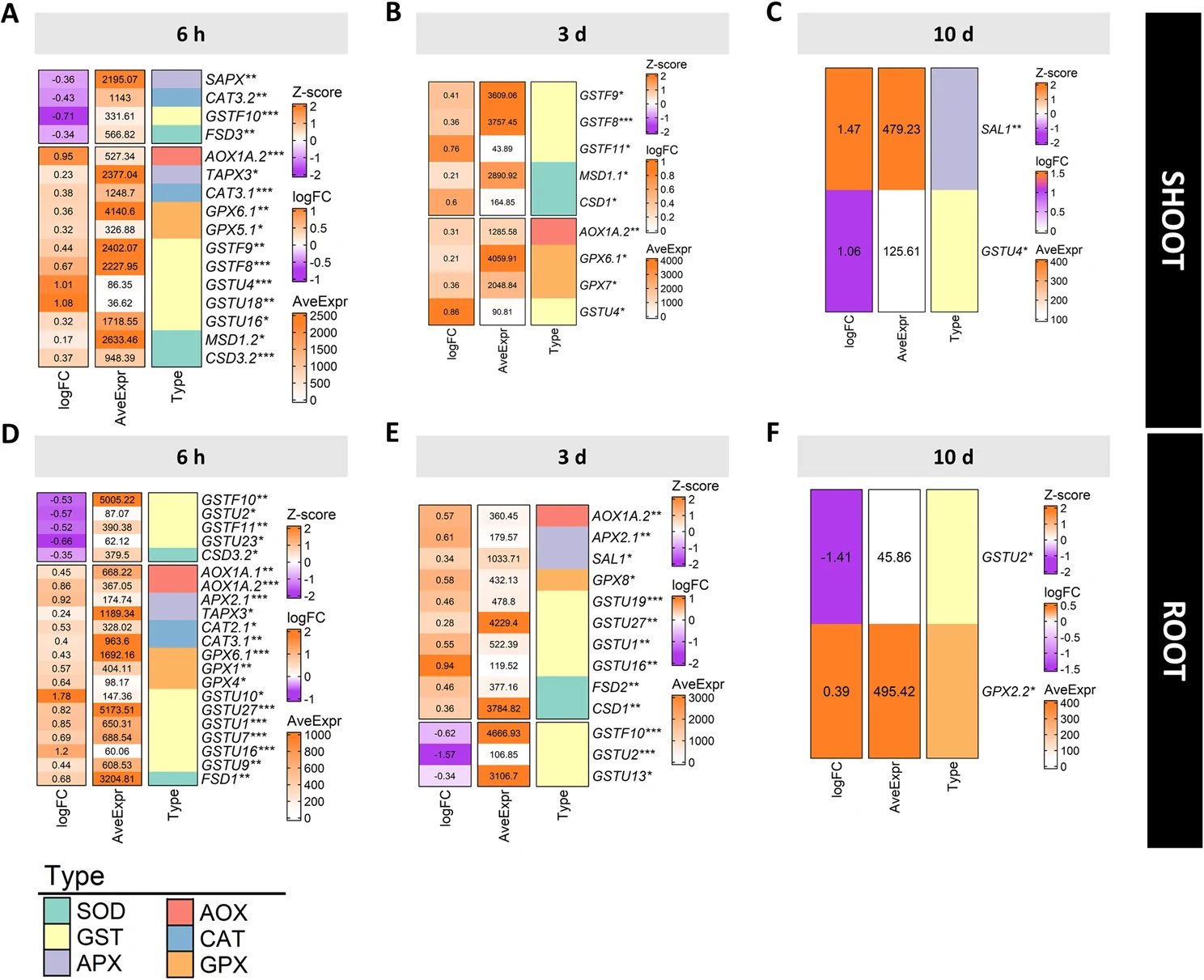
**D**ifferentially expressed genes (DEGs) implicated in oxidative stress response. Antioxidant enzyme-related gene expression in salt-treated and control broccoli plants 6 h (6 h), 3 days (3 d), and 10 days (10 d) after salt application in shoot (**A**, **B**, **C**) and root (**D**, **E**, **F**). Purple and orange indicate negative and positive fold change (logFC), respectively. Mean expression (AveExpr) and gene name are shown. Statistical significance of gene expression differences between salt-treated and control plants was determined based on adjusted p-values (padj) obtained from differential expression analysis (* *p* < 0.05, ** *p* < 0.01, *** *p* < 0.001). Genes shown in the heatmaps are grouped by enzyme type: SOD, superoxide dismutase; GST, glutathione S-transferase; APX, ascorbate peroxidase; AOX, alternative oxidase; CAT, catalase; GPX, glutathione peroxidase

In leaves, DEGs related to oxidative stress response were detected under salinity at all three time points (Fig. 6 and Additional file 8). At 6 h, four genes were downregulated (*SAPX*,* CAT3.2*,* GSTF10*, and *FSD3*), while a marked upregulation was observed for several genes associated with antioxidant defense. Specifically, key antioxidant enzymes such as *GSTF9*, *GSTF8*, *GSTU4*, *GSTU16*, and *GSTU18* were significantly upregulated. Additionally, other oxidative stress-related genes, including *AOX1A.2*, *TAPX3*, *CAT3.1*, *GPX6.1*, and *GPX5.1* were also upregulated (Fig. 6A). At 3 days post-salinity treatment, only upregulated genes were identified in leaf. These included *GSTF9*, *GSTF8*, *GSTF11*, *GSTU4*, *MSD1.1*, *CSD1*, *AOX1A.2*, *GPX6.1*, and *GPX7* (Fig. 6B). By 10 days after salinity application, only one DEG remained: *GSTU4*, which was upregulated with logFC of 1.06 (Fig. 6C).

In the roots, a more diverse set of DEGs was observed at early time points. Several antioxidant-related genes were upregulated, including *AOX1A.2*, *APX2.1*, *TAPX3*, *CAT2.1*, *CAT3.1*, *GPX1*, *GPX4*, and *GPX6.1*, along with multiple GST isoforms such as *GSTU10*, *GSTU27*, *GSTU1*, *GSTU7*, *GSTU16*, and *GSTU9*, as well as the enzyme *FSD1*. Meanwhile, five genes were downregulated, including *GSTF10*, *GSTF11*, *GSTU2*, *GSTU23*, and *CSD3.2* (Fig. 6D). At 3 days, a comparable upregulation pattern was observed, with continued induction of *AOX1A.2*, *APX2.1*, *GPX8*, *GSTU27*, *GSTU1*, *GSTU16*, whereas *GSTF10* (-0.62) and *GSTU2* (-1.57) remained downregulated (Fig. 6E). By 10 days post-treatment, *GPX2.2* showed significant upregulation (logFC of 0.39), while *GSTU2* persisted as the only downregulated gene, with logFC of -1.41 (Fig. 6F).

#### Genes involved in secondary metabolites synthesis related to salt stress response in broccoli plants

##### Glucosinolates

**Fig. 7:**
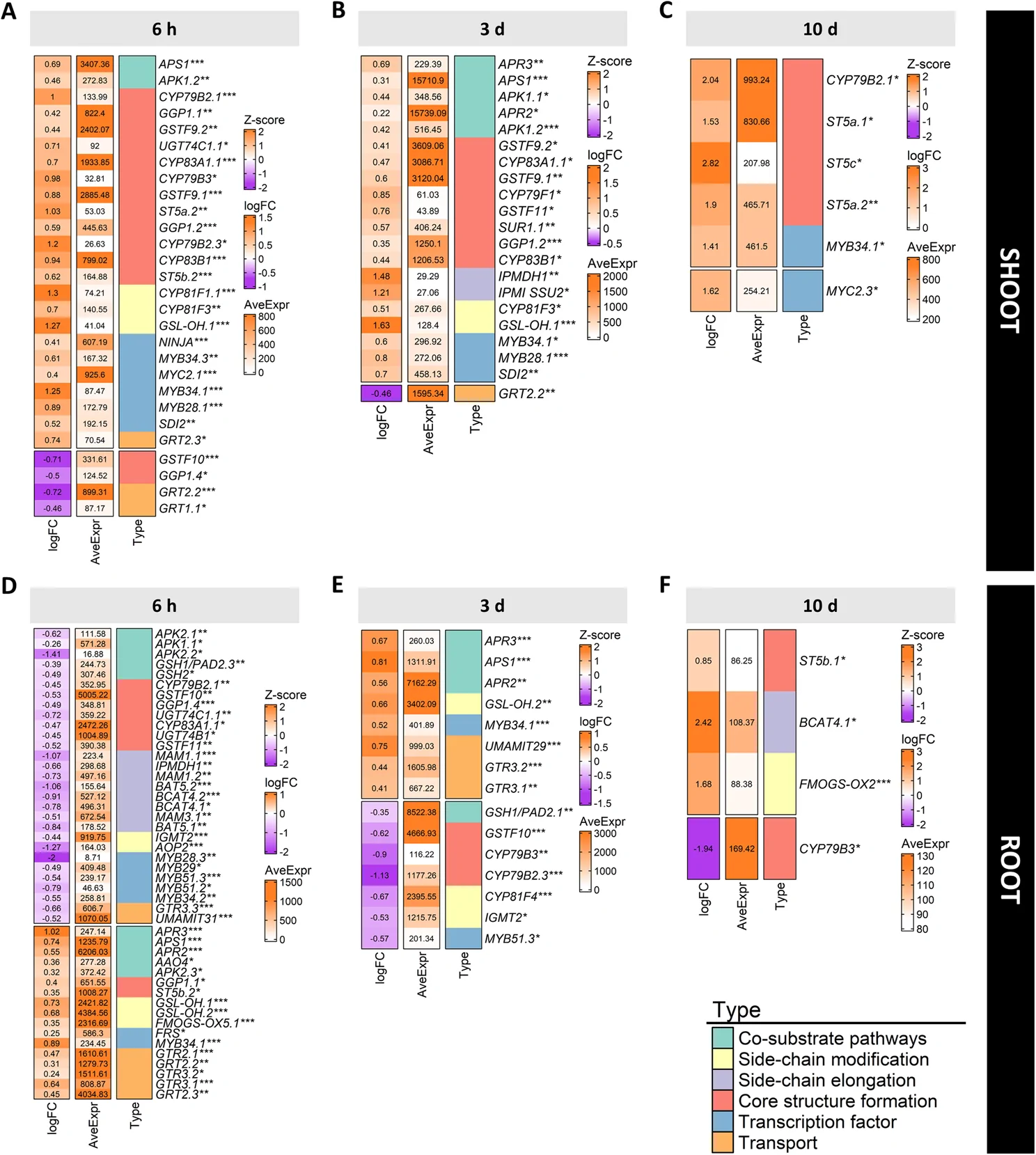
Differentially expressed genes (DEGs) implicated in glucosinolate biosynthesis. Glucosinolate-related gene expression in salt-treated and control broccoli plants 6 h (6 h), 3 days (3 d), and 10 days (10 d) after salt application in shoot (**A**, **B**, **C**) and root (**D**, **E**, **F**). Purple and orange indicate negative and positive fold change (logFC), respectively. Mean expression (AveExpr) and gene name are shown. Statistical significance of gene expression differences between salt-treated and control plants was determined based on adjusted p-values (padj) obtained from differential expression analysis (* *p* < 0.05, ** *p* < 0.01, *** *p* < 0.001). Genes shown in the heatmaps are grouped by gene type: co-substrate pathways, side-chain modification, side-chain elongation, core structure formation, transcription factor, and transport

To assess the impact of salt stress on glucosinolate biosynthesis, we analysed the expression profiles of genes involved in this pathway across multiple functional categories (Fig. 7 and Additional file 9).

In leaf tissues, a marked transcriptional activation was detected as early as 6 h after salt treatment, with broad upregulation of genes involved in core structure formation, side-chain modification, co-substrate pathways, and transcriptional regulation (Fig. 7A). By 3 days, many of these genes maintained elevated expression levels, indicating a sustained response. Notably, the glucosinolate transporter *GTR2.2* remained downregulated at both time points, despite its relatively high basal expression levels (logFC = -0.72 at 6 h and − 0.46 at 3 days) (Fig. 7B). At 10 days post-treatment, the transcriptional activity in leaves was attenuated, with only a few DEGs remaining, including two transcription factors (*MYB34.1* with logFC 1.41 and *MYC2.3* with logFC of 1.62) and a subset of genes related to core structure formation with logFC > 1.4 (Fig. 7C).

In contrast, roots exhibited a more dynamic and complex transcriptional response. At 6 h, both upregulated and downregulated DEGs were detected. Genes involved in side-chain elongation were predominantly repressed, whereas several glucosinolate transporter genes were strongly upregulated, including all three *GTR2* isoforms and two *GTR3* isoforms (Fig. 7D). At 3 days (Fig. 7E), although the number of DEGs declined, reflecting the trend observed in leaves, a dual regulatory pattern persisted. The transcription factor *MYB34.1* remained upregulated (logFC = 0.34), while *MYB51.3* continued to be repressed (logFC=-0.57). Co-substrate pathways-related genes (*APR2*, *APR3*, and *APS1*) showed consistent upregulation across all time points and transporter genes such as *GTR3.1* and *GTR3.2* also remained elevated at 3 days (Fig. 7E).

By 10 days, the transcriptional response in roots further declined, with only a limited number of DEGs remaining. Among them, *BCAT4.1* (side-chain elongation), showed strong induction with logFC of 2.42, whereas *CYP79B3* (core structure formation), remained repressed from 3 days onward, with logFC of − 1.94) (Fig. 7F).

##### Phenylpropanoids

**Fig. 8:**
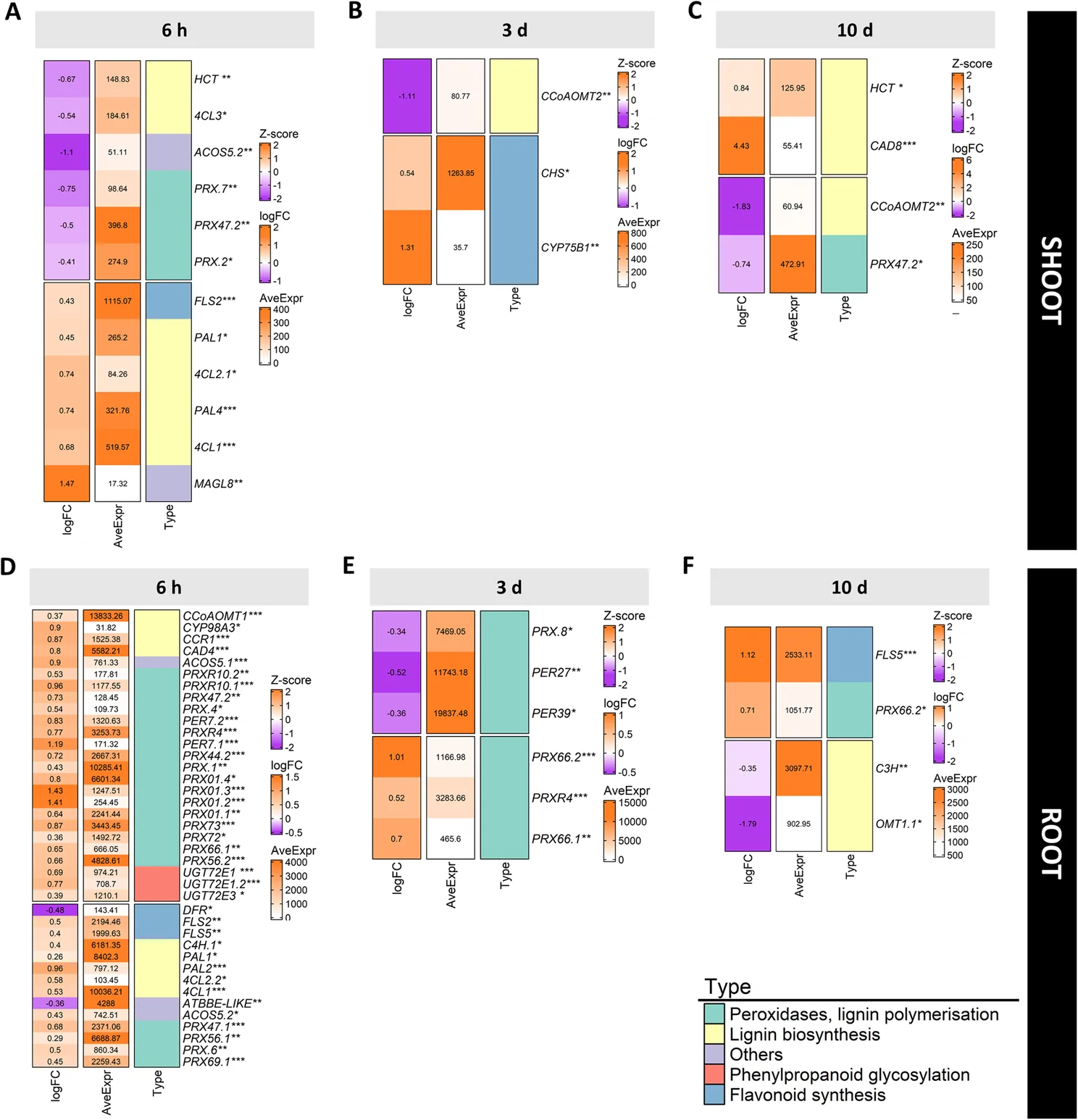
Differentially expressed genes (DEGs) implicated in phenylpropanoid biosynthesis. Phenylpropanoid biosynthesis-related gene expression in salt-treated and control broccoli plants 6 h (6 h), 3 days (3 d), and 10 days (10 d) after salt application in shoot (**A**,** B**, **C**) and root (**D**, **E**, **F**). Purple and orange indicate negative and positive fold change (logFC), respectively. Mean expression (AveExpr) and gene name are shown. Statistical significance of gene expression differences between salt-treated and control plants was determined based on adjusted p-values (padj) obtained from differential expression analysis (* *p* < 0.05, ** *p* < 0.01, *** *p* < 0.001). Genes shown in the heatmaps are grouped by implication in different processes: peroxidases and lignin polymerization, lignin biosynthesis, others related to phenylpropanoids, phenylpropanoids glycosylation, and flavonoid synthesis

The expression profiles of genes involved in the phenylpropanoid biosynthesis pathway were assessed under salinity (Fig. 8 and Additional file 10). The selected genes were related to peroxidases and lignin polymerization, lignin biosynthesis, phenylpropanoid glycosylation, flavonoid synthesis, and other associated processes.

In leaves, at 6 h post-treatment (Fig. 8A), a mixed expression patterns was observed, with several genes, mainly those involved in peroxidase activity and lignin polymerization, being downregulated. These included several class III peroxidases such as *PRX2*, *PRX7*, and *PRX47.2*.

In contrast, genes associated with lignin biosynthesis showed upregulation. At 3 days (Fig. 8B), only three genes were differentially expressed. Notably, two genes involved in flavonoid biosynthesis (*CHS* and *CYP75B1*) were upregulated, while the lignin biosynthesis gene *CCoAOMT2* was downregulated (-1.11), a trend that persisted through 10 days of treatment (-1.83) (Fig. 8C). At this later time point, two other lignin-related genes (*HCT* and *CAD8*) were upregulated instead.

In roots, nearly all selected genes were upregulated at 6 h, particularly class III peroxidases (*PRX*), with several showing strong upregulation (Fig. 8D). Genes involved in phenylpropanoid glycosylation, including three *UGT72E* isoforms, were also upregulated. At 3 days (Fig. 8E), only six genes showed significant expression changes, all belonging to the class III peroxidase family; three were upregulated and three downregulated. However, the downregulated genes exhibited much higher average expression levels, approximately 10-fold greater than those of the upregulated peroxidases. By 10 days (Fig. 8F), only four genes were differentially expressed, including *FLS5* (logFC = 1.12) from the flavonoid synthesis pathway, which was upregulated, and *OMT1.1* (logFC = -1.79) from the lignin biosynthesis pathway, which was downregulated.

## Discussion

**Fig. 9:**
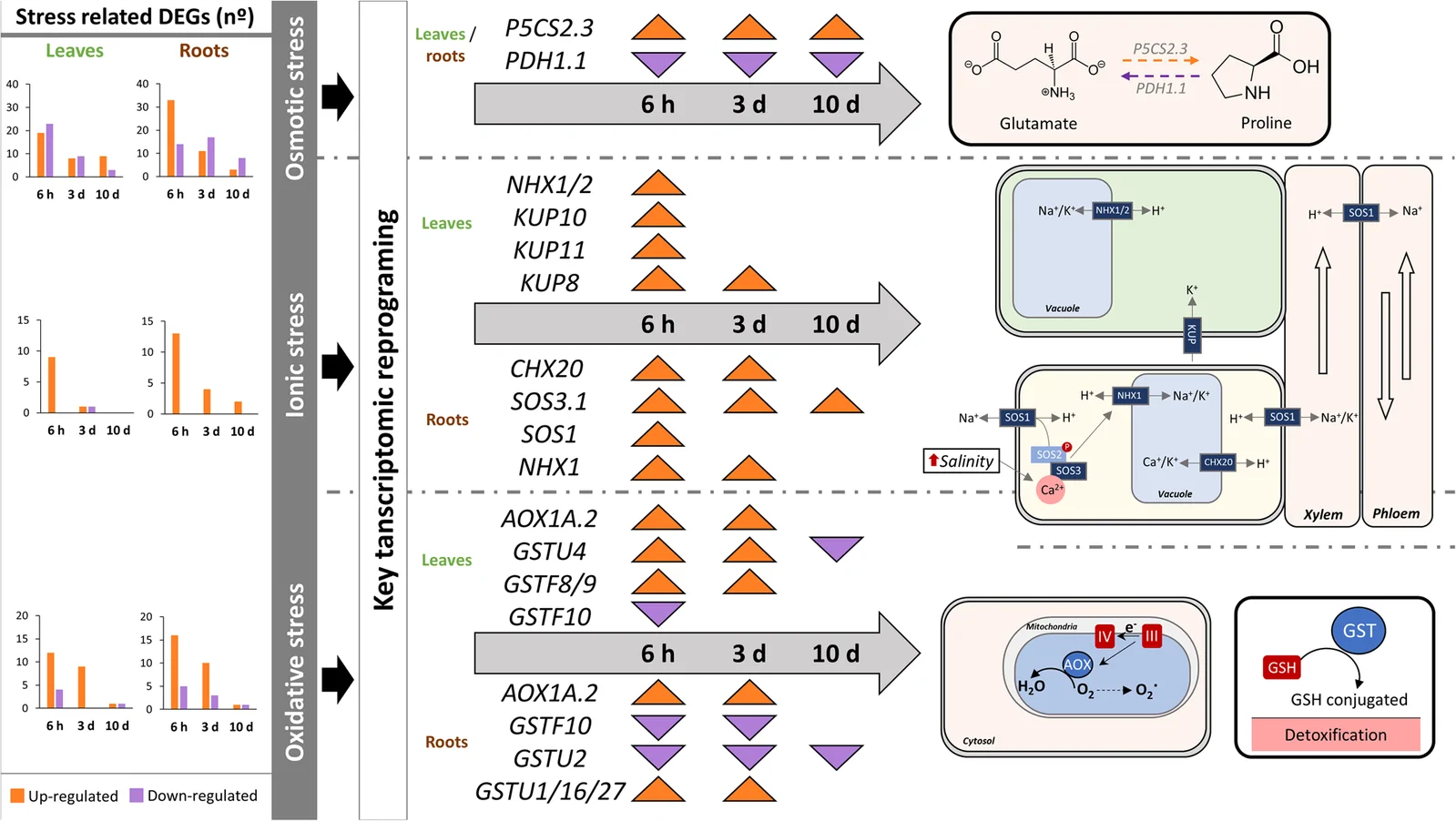
Summary of transcriptomic response of broccoli plants under salt stress. Overview of differentially expressed genes (DEGs) and key stress-responsive pathways in broccoli plants exposed to 80 mM NaCl for 6 h, 3 days, and 10 days. On the left, bar plots represent the number of upregulated (orange) and downregulated (purple) DEGs related to osmotic, ionic, and oxidative stress in both leaf and root tissues. The central schematic summarizes the time-course expression patterns of representative genes involved in proline biosynthesis and degradation, ion transport, and antioxidant activity, with triangle icons indicating the regulation (upregulated, orange; downregulated, purple) and persistence of differential expression over time. The right panel illustrates the main mechanisms triggered under salt stress in broccoli plants: osmotic adjustment via proline accumulation; ionic homeostasis through K⁺ influx, Na⁺ exclusion, and vacuolar compartmentalization; and oxidative stress mitigation via antioxidant enzymes. P5CS, pyrroline-5-carboxylate synthetase; PDH, proline dehydrogenase; KUP, K⁺ uptake permease; NHX, Na⁺/H⁺ exchanger; SOS, salt overly sensitive; CHX, cation/H⁺ exchanger; AOX, alternative oxidase; GST, glutathione S-transferase; GSH, reduced glutathione

While the core strategies that plants employ to cope with salinity are well established, their timing, intensity, and tissue specificity remain poorly characterized in broccoli, a crop for which there is limited research regarding global gene expression changes under salt stress. Recent studies have highlighted how coordinated metabolic and structural adjustments in roots act as key determinants of salinity tolerance in crops [15], underscoring the importance of tissue-specific responses in stress adaptation. In this study, we demonstrate that salt stress in broccoli triggers a complex and highly coordinated network of responses involving hormonal signals, metabolic reprogramming, ion transport, and physiological adjustments, depending on the expression of specific genes (Fig. 9), as well as has been proposed in a recent review [16].

### Physiological and photosynthetic impairments under salt stress

Exposure of broccoli plants to salt stress triggered early and progressive alterations in biomass accumulation, photosynthetic capacity, and CO_2_ exchange parameters. This pattern is consistent with a composite response in which (i) the early phase (first days) is dominated by rapid osmotic and stomatal effects and by acute photochemical impairment, and (ii) the later phase (after several days) reflects partial biochemical acclimation and repair that restores some photosystems capacity but not the throughput of the whole electron transport chain. That salinity produces an early osmotic shock followed by slower ionic toxicity and metabolic costs is a general framework for plant salt responses. The two-phase view (fast osmotic/stomatal effects followed by slower ionic/metabolic effects) is well established for salinity stress [17].

This response reflects growth limitation together with the activation of coordinated adaptive mechanisms, consistent with patterns described for commercial broccoli genotypes under salinity conditions [18, 19].

Interpretation of the gas-exchange and fluorescence data reveals a more complex scenario. At 3 days, the concurrent reductions of A and Ci suggest an early stomatal limitation of CO₂ uptake.

Stomatal closure has been reported to contribute substantially to the initial decline in CO_2_ assimilation [20]. Yet, the simultaneous decline in Fv/Fm, Fv/F₀, and PI at 3 days shows that photosystems photochemistry was also impaired, highlighting that reduced photosynthesis cannot be solely attributed to stomatal limitations. Environmental stresses such as salinity commonly inhibit the repair of photodamaged photosystems, even if direct photodamage is not dramatically higher, which may explain the short-term decrease in fluorescence indices [21]. By day 10, a recovery of Fv/Fm, Fv/F₀, and PI was observed, suggesting a later acclimation of photosystems photochemistry. However, the sustained reduction in ET₀/CS indicates that downstream electron flow remained restricted. This pattern may reflect partial acclimation mediated by antioxidant defense, osmolyte accumulation (e.g., proline), and transcriptome reprogramming, although full recovery of the electron transport chain was not achieved [22]. 

### Osmoprotectant response and ABA coordination under osmotic stress

The most immediate challenge that plants face when subjected to high salinity is the dehydration that results from osmotic stress. To mitigate the increase in osmotic potential in the apoplast, cells initiate osmotic adjustment by accumulating compatible solutes that reduce cellular water potential and maintain turgor [23]. In fact, our previous work already highlighted the relevance of this response level under salt stress in broccoli, as genes related to proline metabolism were among the most enriched in an untargeted transcriptomic approach [12].

This pattern of proline response was associated with an early activation followed by sustained ABA accumulation in roots, supporting its role as a key regulator of osmotic stress responses [4]. The temporal and spatial divergence between roots and leaves suggests that roots prioritize osmotic adjustment through ABA-mediated pathways, while leaves may rely on alternative mechanisms to balance stress responses with photosynthetic activity, which also remained fairly stable during the experiment, reaching levels comparable to those of the control plants after 10 days. The apparent decoupling between ABA levels and physiological outcomes in leaves could be partially explained by the accumulation of SA, which, along with ABA, regulates stomatal closure via distinct ROS signalling pathways. Their interaction can be synergistic or antagonistic depending on timing and environmental conditions [24]. This is consistent with previous reports showing that salinity-induced ABA accumulation is more precise and sensitive in the roots than in leaves of the plant, a phenomenon that has been previously documented in maize plants [25]. In contrast, the absence of ABA accumulation in leaves under our salt conditions suggested a controlled and organ-specific adaptation. The 80 mM NaCl level applied in this study therefore represents a moderate salinity condition, suitable for examining early acclimation mechanisms rather than extreme injury responses [4].

Transcriptomic data corroborated these patterns. Several isoforms of *P5CS*, the key enzyme in proline biosynthesis [26], were upregulated at early time points in roots and leaves remaining overexpressed throughout the experiment. Interestingly, the data suggest that proline accumulation may have been supported not only by the canonical glutamate pathway but also by an alternative route via arginine catabolism. This involves ARGAH-mediated conversion of arginine to ornithine, followed by δ-OAT catalysis of ornithine into glutamate-5-semialdehyde, a direct proline precursor. Although this pathway is not essential under normal growth conditions [27], its early induction under salinity may reflect a rapid mobilization strategy to enhance proline biosynthesis at critical early stages of stress. Additionally, we observed a strong and consistent downregulation across all time points and tissues of proline catabolism genes, particularly *PDH1*, which contributes to proline accumulation by reducing its degradation [26].

These results reinforced the strategy of broccoli plants to maintain high proline pools. Together, the upregulation of biosynthetic routes and repression of catabolic pathways reflects a coordinated regulation of proline homeostasis, supporting its role as a key osmoprotectant and redox buffer during salinity stress. 

### Ionic homeostasis and nutrient imbalance

It has been reported that plants activate mechanisms such as Na^+^ exclusion, vacuolar Na^+^ compartmentalization, and K^+^ retention in the cytosol in response to the accumulation of toxic Na^+^ in plant tissues [28]. In broccoli roots, the early transcriptional activation of K⁺ uptake transporters, particularly members of the KUP family, has been observed. These transporters facilitate K^+^ influx, contributing to initial osmotic adjustment and limiting Na^+^ entry [29]. Nevertheless, their expression declined over time, coinciding with a drop in K^+^ content, suggesting that this strategy is not sustained under prolonged stress. In addition, the moderate fold change and high basal expression of KUP genes may reflect their role in fine-tuning ionic balance rather than executing large-scale ionic shifts.

In contrast, root vacuolar Na^+^ sequestration pathways, including *NHX1.1* and *NHX2*, showed both strong expression and temporal persistence, being upregulated at 6 h and maintained at 3 days. This indicates a strategic shift from initial osmotic protection to long-term ionic detoxification. Accordingly, previous reports on NHX1 showed its involvement in vacuolar Na^+^ compartmentalization in tolerant Brassicas such as *B. rapa* [30]. In fact, the constitutive overexpression of *AtNHX1* has been demonstrated to enhance salinity tolerance [31].

Furthermore, broccoli plants under salinity appear to activate the SOS pathway, which has been shown to improve salt tolerance through ionic adjustments in diverse crops [32]. Particularly *SOS1* at 6 h and the sustained expression *SOS3.1* in roots support a dual mechanism of response: Na⁺ exclusion at the plasma membrane via SOS1 [33], and Na⁺ sequestration into vacuoles via NHX transporters. The persistence of *SOS3.1* expression highlights the importance of this pathway not only for acute stress signalling but also for long-term ionic homeostasis.

A notable finding of our study was the strong and sustained induction of the *CHX20* gene in the roots. However, according to the literature, *CHX20* has primarily been associated with osmoregulatory roles in guard cells [34] and its function in roots remains largely unexplored.

Although an increase in its expression has also been described in the roots of *B. napus* plants 1 h after application of salinity [35]. In line with this early response, a recent transcriptomic study in halophytic plants exposed to extreme salinity levels (up to 600 mM NaCl) has also described the upregulation of CHX family members, including putative *CHX20* homologs, particularly in root tissues [36]. Together, these observations suggest the upregulation of *CHX20* in root tissues under salt stress may be associated with a relevant role in the response of broccoli to salinity.

CHX transporters mediate the exchange of cations, such as K⁺, for H⁺ across cellular membranes, contributing to ion homeostasis and intracellular pH regulation [37]. Activation of *CHX20* could complement the previously described response mechanism, whereby KUP transporters promote K⁺ uptake, and NHX/SOS systems enable Na⁺ exclusion or compartmentalization. In this framework, CHX20 could facilitate these processes by driving proton exchange to maintain favourable electrochemical conditions for ion transport. Sustained *CHX20* expression, particularly in roots, reinforces the concept that this tissue functions as a central site of multi-tiered ionic defense, integrating K⁺ influx, Na⁺ detoxification, and intracellular pH stabilization to mitigate salt-induced cellular damage.

Finally, the response and activated mechanisms are transferred from the roots to the leaves.

Here, the transcriptional response is less pronounced and primarily involves genes from the *KUP* and *NHX* families. *KUP8* stands out for its activation up to three days after treatment, possibly reflecting a localized effort to maintain K⁺ homeostasis in photosynthetic tissues.

*KUP1*, which was also upregulated at 6 h, showed a response comparable to that reported in *B. napus*, where its expression increased one hour later after salt treatment and was linked to cytoplasmic K⁺ maintenance and turgor regulation under stress [35].

Additionally, the upregulation of two *NHX1* isoforms suggested an enhancement of defense mechanisms to sequester incoming Na⁺ into vacuoles in shoots. The upregulation of this gene is similar to what has been described in other salt-stressed species [38], where it has been described as key to achieving salt tolerance through ionic homeostasis. However, this response is not sustained over time and is therefore likely important in the initial response. In fact, ten days later, no significant differential expression of major Na⁺ or K⁺ transporters was detected in shoots, which could suggest either a transition towards passive ion management or that the energetic cost of active transport is no longer sustainable.

Together with ion toxicity, salinity also disrupts nutrient homeostasis, either through competition between Na^+^ and essential cations or by impairing root function and transporter activity. In leaf tissue, the decrease in essential macronutrients is related to the metabolic imbalances found in plants subjected to salinity, such as decreased biomass and photosynthesis.

However, a different profile was observed in the roots, which may be focused on enhancing the activity of enzymes involved in ROS detoxification. The sustained levels of Zn, Fe, and Mn (Additional file 4), cofactors of these enzymes, under stress conditions, may help reinforce their activity, alongside the upregulation of several *CAT*, *GSTU* (glutathione S-transferases, tau class) and *GPX* (glutathione peroxidase) enzyme genes [39]. This mechanism reinforces the idea that roots not only serve as sensors but also as metabolic centres for oxidative stress mitigation. Moreover, when analysing several transcription factors, we observed an early repression (6 h) of *bZIP23.1* (Additional file 11), that has been directly related with Zn uptake [40]. 

### Enzymatic response to oxidative stress

Salt stress induces a rapid accumulation of ROS, leading to oxidative damage unless counteracted by efficient antioxidant responses [41]. Transcriptomic analysis revealed a dynamic, tissue- and time-dependent upregulation of antioxidant-related genes, particularly at early stages. These included *AOX*, *APX*, *CAT*, *GPX*, and a broad range of *GSTU* isoforms, which are key enzymatic components of the ROS detoxification network [41]. Similar expression patterns have been reported in *Arabidopsis* and *Brassica* species, including salt-tolerant varieties [42].

Notably, in roots, *GSTU* genes formed the largest group of differentially expressed antioxidant-related transcripts, with several isoforms showing strong upregulation during the early phase, suggesting the essential role of roots in managing early ROS increases. These enzymes play dual roles in ROS detoxification and xenobiotic conjugation, and their sustained expression has suggested a potential requirement for ROS buffering and redox homeostasis beyond the initial stress signal [43]. For example, despite its low basal expression, *GSTU4* exhibited strong and sustained upregulation throughout our experiment, which suggests a protective role against lipid peroxidation and detoxifies toxic metabolites generated during both the acute and prolonged phases of salt stress in broccoli. Therefore, this gene has been linked to the adaptation of plants to salt stress [43, 44].

Another key antioxidant gene upregulated in our study was *AOX1A.2*, which is involved in limiting mitochondrial ROS production. Its overexpression may reflect an adjustment to maintain mitochondrial function under stress conditions, as it has been previously observed [45]. Furthermore, *GPX6.1* showed strong upregulation in both roots and leaves just 6 h after salt treatment, indicating a rapid and systemic antioxidant response related genes. This early increased expression suggested an important role for GPX6 in the immediate containment of ROS across all tissues. In contrast, *GPX2.2* was specifically upregulated in roots at 10 days, which may reflect a prolonged state of oxidative stress and the need to maintain membrane integrity during later phases of stress. This differential temporal and spatial regulation of *GPX* isoforms supports the existence of a coordinated antioxidant response deployed by broccoli under salinity conditions. According to this, previous findings in *Brassica* species, showed increased *GPX* expression has been associated with membrane integrity maintenance under salt stress [46]. Altogether, our results reveal a multi-layered antioxidant defense in broccoli, with root-specific early activation of *GSTs* and *AOX*, and systemic involvement of *GPX* isoforms over time. However, further validation of these transcriptomic responses would benefit from complementary analyses, including enzyme activity measurements and direct assessment of ROS levels across tissues and time points.

Hormonal changes likely contributed to this transcriptional response, although this targeted hormone panel should be interpreted as a complementary layer rather than as a comprehensive hormonal analysis. The selected compounds included representative stress-related hormones, such as ABA, SA and JA-related compounds, together with growth-associated markers, such as IAA and zeatin, allowing a partial assessment of the balance between stress signalling and growth regulation [14, 47]. However, other important growth-related regulators, such as gibberellins, brassinosteroids and strigolactones, were not quantified, their inclusion in future studies would provide a more comprehensive view of hormonal regulation under salinity. In leaves, the transient increase in SA observed may have acted as an early alarm signal to stimulate antioxidant enzyme expression, as SA has been shown to activate antioxidant defense under osmotic and oxidative stress [48]. This initial response can prepare the plant or modulate other signals (JA, ROS, or ABA). In addition, auxin-related responses also showed a coordinated pattern at later stages of stress. The observed decrease in IAA levels at 3 and 10 days, particularly in leaves, together with the repression of several SAUR genes (Additional file 12), suggests a downregulation of auxin-mediated growth processes under salinity. Given the role of SAUR proteins in promoting cell expansion through activation of plasma membrane H⁺-ATPases [49], this response likely reflects a shift from growth to stress adaptation in shoots.

In roots, we observed a general short-term accumulation of several measured hormones, which then decreased over time. This suggests that early hormonal signalling in the roots was critical not only for mitigating local stress, but also for initiating systemic responses and activating signalling cascades towards leaves in broccoli plants. 

### Non-enzymatic response to oxidative stress

In addition to enzymatic mechanisms, plants display a range of non-enzymatic responses to mitigate oxidative stress, including the synthesis of phenolic compounds [50]. Interestingly, the activation of flavonoid biosynthetic genes (*CHS*,* FLS5*,* CYP75B1*) at 3 and 10 days post-treatment suggests that non-enzymatic antioxidant defense also contributes to ROS mitigation in shoots. Moreover, we detected a significant increase in chlorogenic acid at 10 days. Phenolics are known to scavenge ROS in chloroplasts and vacuoles, stabilize membranes, and modulate auxin transport and hormonal signalling, making them versatile compounds for long-term protection [50]. Therefore, the observed recovery of Fv/Fm, Fv/F₀ and PI by 10 days, suggested that the photosynthetic apparatus could be protected by chlorogenic acid [51]. However, the integrated electron transport through the chain remained constrained since the nutrient imbalance and toxicity was still present.

GSLs are a major class of secondary metabolites in *Brassica* species with recognized roles in stress signalling and plant defense [52]. Although it is generally reported that salinity induces an increase in GSL content, this response varies considerably depending on the species, stress intensity, cultivar, and even seasonal factors [53]. Our metabolite profiling revealed a decrease in glucobrassicin content in leaves at 10 days, which contrasts with the transcriptional upregulation of GSL biosynthesis genes observed mainly in shoots. This apparent discrepancy suggests that post-transcriptional regulation or turnover of GSLs may be at play under salt stress conditions.

Moreover, it is essential to consider that GSLs are enzymatically hydrolysed into isothiocyanates and other bioactive breakdown products by the action of myrosinase, an enzyme typically localized in the cytoplasm, whereas GSLs are stored in vacuoles. Under salinity stress, membrane damage may increase the contact between GSLs and myrosinase, thus promoting GSL degradation and the release of defense compounds [8]. It is important to note that the reduction in glucobrassicin does not necessarily reflect deficient biosynthesis. Rather, it may reflect enhanced hydrolytic turnover, which could contribute to antioxidant protection, modulation of stress-related signalling networks, and interactions with hormonal pathways [54].

Apart from the synthesis and degradation pathways, the transport and remobilization of GSLs between different tissues must play a fundamental role in the response to salinity. In this regard, our transcriptomic analysis revealed tissue-specific regulation of GTRs, which has been previously reported in other *Brassica* species [9]. We found repression of *GTR2* in leaves and activation of *GTR* genes in roots, suggesting a redistribution of GSLs towards the roots. Thus, this differential regulation supports the notion that the roots, as the primary site of salt perception and early stress signalling, may act as a focal point for GSL mediated stress responses. In this context, GSL accumulation and turnover in roots may contribute to local stress responses by generating bioactive breakdown products that modulate ROS-related signalling pathways and activate antioxidant defense.

In conclusion, this study provides a spatiotemporal and multi-omic map of the broccoli response to moderate salt stress. It reveals a coordinated, tissue-specific strategy involving deep systemic communication that synchronizes stress detection in the roots with metabolic adjustments in the leaves (Fig. 9). Roots act as primary stress sensors, activating early signalling cascades that included ionic exclusion mechanisms and osmoprotection, driven by the induction of key transporters, including KUP family genes for K⁺ uptake, *NHX1.1* and *NHX2* for vacuolar Na⁺ sequestration, and *SOS1/SOS3.1* for Na⁺ efflux into the apoplast. Notably, the root-specific induction of *CHX20*, highlights a prioritized mechanism for pH homeostasis and Na⁺ detoxification. This is further supported by the rapid activation of proline biosynthesis via P5CS and the simultaneous repression of the catabolic gene *PDH1*, ensuring a stable osmoprotective pool. In contrast, leaves implement delayed but effective metabolic shifts to preserve photosynthetic function. This coordination is driven by a complex hormonal redistribution, involving ABA, JA, and IAA accumulation in roots and early SA increases in shoots. Oxidative stress management was mediated by strong early activation of antioxidant enzyme genes such as *AOX*, *SOD*, *GPX*, and multiple *GST* isoforms in roots, along with delayed activation of flavonoid biosynthesis genes (*CHS, FLS5, CYP75B1*) and chlorogenic acid accumulation in leaves.

From an applied perspective, these results provide a framework for understanding acclimation to moderate salinity in broccoli and may help identify physiological and molecular processes relevant for improving stress resilience in *Brassica* crops. Targeting the temporal and organ-specific efficiency of root-driven ion transport and leaf secondary metabolism represents a promising strategy to sustain agricultural productivity under increasingly saline conditions.

However, comparative studies including contrasting genotypes and broader salinity gradients will be required to validate their potential use in breeding strategies. 

## Supplementary Information

Supplementary Material 1: Additional file 1. Total fresh weight (FW) of broccoli plants under control (C) conditions and after 6 hours, 3 days and 10 days after application of salinity stress (NaCl). Additional file 2. Principal Component Analysis (PCA) of micro and macronutrients in (A) leaves and (B) roots of broccoli plants under control (C) conditions and after 6 hours, 3 days and 10 days after application of salinity stress (NaCl). Additional file 3. Analysis of mineral nutrients in leaves of broccoli plants under control (C) conditions and after 6 hours, 3 days and 10 days after application of salinity stress (NaCl). Additional file 4. Analysis of mineral nutrients in roots of broccoli plants under control (C) conditions and after 6 hours, 3 days and 10 days after application of salinity stress (NaCl). Additional file 5. Summary of differentially expressed genes (DEGs) categorized by functional stress response. Additional file 6. Heatmaps of gene expression related to osmolyte biosynthesis in broccoli plants subjected to salt treatment and control conditions at 6 hours (6h), 3 days (3d), and 10 days (10d) after salt application, in leaves (A, C, E) and root (B, D, F). Additional file 7. Heatmaps of gene expression related to ionic stress response in broccoli plants subjected to salt treatment and control conditions at 6 hours (6h), 3 days (3d), and 10 days (10d) after salt application, in leaves (A, B, C) and root (D, E, F). Additional file 8. Heatmaps of gene expression related antioxidant enzyme in broccoli plants subjected to salt treatment and control conditions at 6 hours (6h), 3 days (3d), and 10 days (10d) after salt application, in leaves (A, C, E) and root (B, D, F). Additional file 9. Heatmaps of gene expression related to glucosinolates in broccoli plants subjected to salt treatment and control conditions at 6 hours (6h), 3 days (3d), and 10 days (10d) after salt application, in leaves (A, C, E) and root (B, D, F). Additional file 10. Heatmaps of gene expression related to phenylpropanoid biosynthesis in broccoli plants subjected to salt treatment and control conditions at 6 hours (6h), 3 days (3d), and 10 days (10d) after salt application, in leaves (A, C, E) and root (B, D, F). Additional file 11. Heatmaps of gene expression of transcription factors (TF) in broccoli plants subjected to salt treatment and control conditions at 6 hours (6h), 3 days (3d), and 10 days (10d) after salt application, in leaves (A, B, C) and root (D, E, F). Additional file 12. Heatmaps of gene expression related to auxin biosynthesis in broccoli plants subjected to salt treatment and control conditions at 6 hours (6h), 3 days (3d), and 10 days (10d) after salt application, in leaves (A, C, E) and root (B, D, F).

## Data Availability

The omics dataset generated and analysed in this study has been deposited in the NCBI database under accession number PRJNA1327109. Additional datasets supporting the conclusions of this work are available from the corresponding author upon reasonable request.
