# Supplementary material for: Whole-plant spatiotemporal responses to salinity reveal osmotic and ionic drivers of salt tolerance in broccoli
Source: BMC Plant Biol. 2026 Jun 30;26:1430. doi: 10.1186/s12870-026-09390-0 (PMC13491636; doi:10.1186/s12870-026-09390-0)
Supplement: Supplementary file 1 [file 12870_2026_9390_MOESM1_ESM.docx]

**Additional file 1.** Total fresh weight (FW) of broccoli plants under control (C) conditions and after 6 hours, 3 days and 10 days after application of salinity stress (NaCl). Data is represented as boxes (25–75%), error bars represent the range within a 1.5 quartile and the median line (n = 6). Asterisks mean statistical differences after T-Student test (*p < 0.05, **p < 0.01, ***p < 0.001).

**Additional file 2.** Principal Component Analysis (PCA) of micro and macronutrients in **(A)** leaves and **(B)** root of broccoli plants under control conditions and after 6 hours, 3 days and 10 days after application of salinity stress (NaCl), with arrows indicating loadings of each nutrient; confidence ellipses were plotted for each treatment, each small circle represents the data from an individual sample. The data are represented as a whole and separating the samples from a temporal point of view (6 hours, 3 days and 10 days).

**Additional file 3.** Analysis of mineral nutrients in leaves of broccoli plants under control (C) conditions and after 6 hours, 3 days and 10 days after application of salinity stress (NaCl). Data is represented as boxes (25–75%), error bars represent the range within a 1.5 quartile and the median line (n = 6). Asterisks mean statistical differences after T-Student test (*p < 0.05, **p < 0.01, ***p < 0.001).

**Additional file 4.** Analysis of mineral nutrients in root of broccoli plants under control (C) conditions and after 6 hours, 3 days and 10 days after application of salinity stress (NaCl). Data is represented as boxes (25–75%), error bars represent the range within a 1.5 quartile and the median line (n = 6). Asterisks mean statistical differences after T-Student test (*p < 0.05, **p < 0.01, ***p < 0.001).

**Additional file 5**. Summary of differentially expressed genes (DEGs) categorized by functional stress response. The table shows the total number of up-regulated and down-regulated genes related to osmotic, ionic, and oxidative stress in both shoot and root tissues at three time points (6 hours, 3 days, and 10 days) after salinity exposure. DEGs were identified based on an adjusted p-value (q < 0.05).

|  |  | | **Number of Differentially Expressed Genes (DEGs)** | | | | | | | |
| --- | --- | --- | --- | --- | --- | --- | --- | --- | --- | --- |
|  | | **Shoot** | | | | | **Root** | | | |
|  | | **Total** | | **% of DEGs ^a^** | **Up** | **Down** | **Total** | **% of DEGs ^a^** | **Up** | **Down** |
| **Osmotic stress** | | | | | | | | | | |
| 6 hours | | 42 | | 3.5% | 19 | 23 | 47 | 7.0% | 33 | 14 |
| 3 days | | 17 | | 11.2% | 8 | 9 | 28 | 8.7% | 11 | 17 |
| 10 days | | 12 | | 2.2% | 9 | 3 | 11 | 4.0% | 3 | 8 |
|  | | **71** | | **3.7%** | **36** | **35** | **86** | **6.8%** | **47** | **39** |
| Ionic stress | | | | | | | | | | |
| 6 hours | | **9** | | 0.7% | 9 | 0 | **13** | 1.9% | 13 | 0 |
| 3 days | | **2** | | 1.3% | 1 | 1 | **4** | 1.2% | 4 | 0 |
| 10 days | | **0** | | 0.0% | 0 | 0 | **2** | 0.7% | 2 | 0 |
|  | | **11** | | **0.6%** | **10** | **1** | **19** | **1.5%** | **19** | **0** |
| Oxidative stress | | | | | | | | | | |
| 6 hours | | **16** | | 1.3% | 12 | 4 | **21** | 3.1% | 16 | 5 |
| 3 days | | **9** | | 5.9% | 9 | 0 | **13** | 4.0% | 10 | 3 |
| 10 days | | **2** | | 0.4% | 1 | 1 | **2** | 0.7% | 1 | 1 |
|  | | **27** | | **1.4%** | **22** | **5** | **36** | **2.8%** | **27** | **9** |
| **Cumulative Total**  **(all times)** | | **109** | | **5.7%** ᵇ |  |  | **141** | **11.1%** ᵇ |  |  |

^a^ Time-points percentages were calculated relative to the total number of differentially expressed genes (DEGs) identified at each specific time point in our previous study [12]. Specifically, percentages are based on a total of 1207, 152, and 555 DEGs for shoots, and 667, 322, and 277 DEGs for roots at 6 hours, 3 days, and 10 days, respectively.

ᵇ Cumulative percentages were calculated relative to the grand total of unique DEGs identified across the entire experiment (1914 total DEGs for shoots and 1266 total DEGs for roots) [12].

**Additional file 6.** Heatmaps of gene expression related to osmolyte biosynthesis in broccoli plants subjected to salt treatment and control conditions at 6 hours (6h), 3 days (3d), and 10 days (10d) after salt application, in leaves **(A, C, E)** and root **(B, D, F)**. Purple indicates low expression and orange high expression (Z-score), fold change (logFC), mean expression (AveExpr), and gene name are shown. Statistical significance of gene expression differences between salt-treated and control plants was determined based on adjusted p-values (padj) obtained from differential expression analysis (*p < 0.05, **p < 0.01, ***p < 0.001). Genes shown in the heatmaps are grouped by the different pathways involved.

**Additional file 7.** Heatmap of ion transport-related gene expression in salt-treated and control broccoli plants 6 hours (6h), 3 days (3d), and 10 days (10d) after salt application in leaves (**A, B, C**) and root (**D, E, F**). Purple represents genes with low expression, orange represents genes with high expression (Z-score), fold change (logFC), mean expression (AveExpr), and gene name are shown. Statistical significance of gene expression differences between salt-treated and control plants was determined based on adjusted p-values (padj) obtained from differential expression analysis (*p < 0.05, **p < 0.01, ***p < 0.001). Genes shown in the heatmaps are grouped by gene type: CHX, cation/H+ - exchanger; HKT, high-affinity K+ transporter; HAK/KUP/KT, high-affinity K+ transporters/K+ uptake permeases/K+ transporters; NHX, sodium/hydrogen antiporter; SOS, salt overly sensitive proteins.

**Additional file 8.** Heatmap of antioxidant enzyme-related gene expression in salt-treated and control broccoli plants 6 hours (6h), 3 days (3d), and 10 days (10d) after salt application in leaves (A, C, E) and root (B, D, F). Purple represents genes with low expression, orange represents genes with high expression (Z-score), fold change (logFC), mean expression (AveExpr), and gene name are shown. Statistical significance of gene expression differences between salt-treated and control plants was determined based on adjusted p-values (padj) obtained from differential expression analysis (*p < 0.05, **p < 0.01, ***p < 0.001). Genes shown in the heatmaps are grouped by enzyme type: SOD, superoxide dismutase; GST, glutathione S-transferase; APX, ascorbate peroxidase; AOX, alternative oxidase; CAT, catalase; GPX, glutathione peroxidase.

**Additional file 9.** Heatmap of glucosinolates-related gene expression in salt-treated and control broccoli plants 6 hours (6h), 3 days (3d), and 10 days (10d) after salt application in leaves (A, C, E) and root (B, D, F). Purple represents genes with low expression, orange represents genes with high expression (Z-score), fold change (logFC), mean expression (AveExpr), and gene name are shown. Statistical significance of gene expression differences between salt-treated and control plants was determined based on adjusted p-values (padj) obtained from differential expression analysis (*p < 0.05, **p < 0.01, ***p < 0.001). Genes shown in the heatmaps are grouped by gene type: co-substrate pathways, side-chain modification, side-chain elongation, core structure formation, transcription factor, and transport.

**Additional file 10.** Heatmap of phenylpropanoid biosynthesis-related gene expression in salt-treated and control broccoli plants 6 hours (6h), 3 days (3d), and 10 days (10d) after salt application in leaves (A, C, E) and root (B, D, F). Purple represents genes with low expression, orange represents genes with high expression (Z-score), fold change (logFC), mean expression (AveExpr), and gene name are shown. Statistical significance of gene expression differences between salt-treated and control plants was determined based on adjusted p-values (padj) obtained from differential expression analysis (*p < 0.05, **p < 0.01, ***p < 0.001). Genes shown in the heatmaps are grouped by implication in different processes: peroxidases and lignin polymerization, lignin biosynthesis, others related to phenylpropanoids, phenylpropanoids glycosylation, and flavonoid synthesis.

**Additional file 11.** Heatmaps of gene expression of transcription factors (TF) in broccoli plants subjected to salt treatment and control conditions at 6 hours (6h), 3 days (3d), and 10 days (10d) after salt application, in leaves **(A, B, C)** and root **(D, E, F)**. Purple indicates low expression and orange high expression (Z-score), fold change (logFC), mean expression (AveExpr), and gene name are shown. Statistical significance of gene expression differences between salt-treated and control plants was determined based on adjusted p-values (padj) obtained from differential expression analysis (*p < 0.05, **p < 0.01, ***p < 0.001). Genes displayed in the heatmaps are grouped according to transcription factor families: AP2/ERF (APETALA2/Ethylene Responsive Factor), bHLH (basic helix–loop–helix), bZIP (basic leucine zipper), HDZip (homeodomain–leucine zipper), MYB (myeloblastosis family), NAC (NAM, ATAF1/2 and CUC2), NF-Y (Nuclear Factor Y), WRKY (WRKY transcription factors), and ZF (zinc finger).

**Additional file 12.** Heatmap of auxin biosynthesis-related gene expression in salt-treated and control broccoli plants 6 hours (6h), 3 days (3d), and 10 days (10d) after salt application in leaves (A, C, E) and root (B, D, F). Purple represents genes with low expression, orange represents genes with high expression (Z-score), fold change (logFC), mean expression (AveExpr), and gene name are shown. Statistical significance of gene expression differences between salt-treated and control plants was determined based on adjusted p-values (padj) obtained from differential expression analysis (*p < 0.05, **p < 0.01, ***p < 0.001). Genes shown in the heatmaps are grouped by gene type: GH3, Gretchen Hagen 3; SAUR, Small Auxin-Upregulated RNA; IAA, Indole-3-acetic acid-induced proteins; YUCCA, Flavin monooxygenases involved in auxin biosynthesis; PIN, PIN-FORMED auxin efflux carriers; TAA+TAR, Tryptophan aminotransferases involved in auxin biosynthesis; ARF, Auxin Response Factors; AUX1-LAX, Auxin influx carriers; TIR+AFB, Transport Inhibitor Response1 / Auxin Signalling F-Box proteins.
